# A metagenomic viral discovery approach identifies potential zoonotic and novel mammalian viruses in *Neoromicia* bats within South Africa

**DOI:** 10.1371/journal.pone.0194527

**Published:** 2018-03-26

**Authors:** Marike Geldenhuys, Marinda Mortlock, Jacqueline Weyer, Oliver Bezuidt, Ernest C. J. Seamark, Teresa Kearney, Cheryl Gleasner, Tracy H. Erkkila, Helen Cui, Wanda Markotter

**Affiliations:** 1 Centre for Viral Zoonoses, Department of Medical Virology, University of Pretoria, Pretoria, South Africa; 2 Centre for Emerging Zoonotic and Parasitic Diseases, National Institute for Communicable Diseases, National Health Laboratory Services, Johannesburg, South Africa; 3 Centre for Microbial Ecology and Genomics, University of Pretoria, Pretoria, South Africa; 4 AfricanBats NPC, Pretoria, South Africa; 5 Eugène Marais Chair of Wildlife Management, Mammal Research Institute, University of Pretoria, Pretoria, South Africa; 6 Ditsong National Museum of Natural History, Pretoria, South Africa; 7 School of Animal, Plant and Environmental Sciences, University of the Witwatersrand, Johannesburg, South Africa; 8 Los Alamos National Laboratory, Los Alamos, New Mexico, United States of America; Deutsches Primatenzentrum GmbH - Leibniz-Institut fur Primatenforschung, GERMANY

## Abstract

Species within the *Neoromicia* bat genus are abundant and widely distributed in Africa. It is common for these insectivorous bats to roost in anthropogenic structures in urban regions. Additionally, *Neoromicia capensis* have previously been identified as potential hosts for Middle East respiratory syndrome (MERS)-related coronaviruses. This study aimed to ascertain the gastrointestinal virome of these bats, as viruses excreted in fecal material or which may be replicating in rectal or intestinal tissues have the greatest opportunities of coming into contact with other hosts. Samples were collected in five regions of South Africa over eight years. Initial virome composition was determined by viral metagenomic sequencing by pooling samples and enriching for viral particles. Libraries were sequenced on the Illumina MiSeq and NextSeq500 platforms, producing a combined 37 million reads. Bioinformatics analysis of the high throughput sequencing data detected the full genome of a novel species of the *Circoviridae* family, and also identified sequence data from the *Adenoviridae*, *Coronaviridae*, *Herpesviridae*, *Parvoviridae*, *Papillomaviridae*, *Phenuiviridae*, and *Picornaviridae* families. Metagenomic sequencing data was insufficient to determine the viral diversity of certain families due to the fragmented coverage of genomes and lack of suitable sequencing depth, as some viruses were detected from the analysis of reads-data only. Follow up conventional PCR assays targeting conserved gene regions for the *Adenoviridae*, *Coronaviridae*, and *Herpesviridae* families were used to confirm metagenomic data and generate additional sequences to determine genetic diversity. The complete coding genome of a MERS-related coronavirus was recovered with additional amplicon sequencing on the MiSeq platform. The new genome shared 97.2% overall nucleotide identity to a previous *Neoromicia*-associated MERS-related virus, also from South Africa. Conventional PCR analysis detected diverse adenovirus and herpesvirus sequences that were widespread throughout *Neoromicia* populations in South Africa. Furthermore, similar adenovirus sequences were detected within these populations throughout several years. With the exception of the coronaviruses, the study represents the first report of sequence data from several viral families within a Southern African insectivorous bat genus; highlighting the need for continued investigations in this regard.

## Introduction

The role of bats as potential or confirmed reservoirs of various viral agents with public health importance has been increasingly appreciated in recent years. Several bat-borne viruses are considered emerging, with an increase in the number of human cases and outbreaks over the past two decades; such as the bat-borne viruses Marburg virus and Nipah virus [1,2]. These viruses are associated with direct zoonotic transmission and infection of exposed human populations. Other identified bat-associated viruses are only related to viruses of known public health importance, like the severe acute respiratory syndrome coronavirus (SARS-CoV) or Middle East respiratory syndrome coronavirus (MERS-CoV) [3–5]. Though no direct spillover have been identified, overall sequence similarities between human viral strains and related bat-borne viruses have implicated these viruses in the emergence of new human coronaviruses [6,7]. Detection of potentially zoonotic viruses in bat species has been the driving force for increased research and surveillance of the bat virome (as well as other infectious agents).

Viral discovery with conventional nucleic acid detection assays targeting conserved gene regions (such as for PCR-based surveillance) as well as viral metagenomic studies have been successful in analysing the viral richness harboured by bats [8–14]. The use of sequence-independent metagenomic approaches has enabled detection of highly divergent viruses, often sharing only low homologies to currently known virus species [11,14–17]. Viral metagenomic investigations provide opportunities for the detection of potentially zoonotic viruses, as well as other mammalian-infecting viruses not previously known to have been present [8,11,18]. An inventory of novel viruses can thus be constructed, and further investigated for their potential as zoonotic agents. Metagenomics and conventional PCR surveillance have identified bats as natural hosts for large genetic diversities of several viral families—such as the *Circoviridae*, *Coronaviridae*, *Paramyxoviridae*, *Parvoviridae*, and *Adenoviridae*, as well as the genus *Hepacivirus*, in the family *Flaviviridae* [6,10,19,20].

Insectivorous bats of the genus *Neoromicia* (family Vespertilionidae) referred to commonly as either Serotine or Pipistrelle bats, are distributed in the Afro-Malagasy regions. Thirteen of the seventeen currently recognized species occur on mainland Africa, with six species (*Neoromicia capensis*, *N*. *nana*, *N*. *rendalli*, *N*. *stanleyi*, *N*. *zuluensis* and *N*. *cf*. *helios*) recorded in South Africa [21,22]. *Neoromicia* bats habitually roost in small colony numbers (up to 10 individuals) in naturally occurring crevices, though some species may also be found in anthropogenic structures such as the roofs of houses [21].

The International Union for Conservation of Nature (IUCN) Red List conservation status varies per species. *Neoromicia nana* and *N*. *capensis* are both considered “Least Concern” (i.e. not threatened) in the global IUCN Red List assessment due to their wide geographic range and abundance, with no population declines currently known [23,24]. Investigations have identified that both species host various viruses. The novel hantavirus, Mouyassué virus, was detected from archival specimens of two *N*. *nana* individuals collected in the Ivory Coast in West Africa [25]. In South Africa, novel paramyxovirus sequences were identified from the same host species [26]. Lastly, coronaviruses from both the genera *Alphacoronavirus* and *Betacoronavirus* have been reported from *N*. *capensis* species [27,28]. A detected lineage C betacoronavirus, named NeoCoV/PML-PHE1, grouped taxonomically within the same species as MERS-CoV [4,28]. The virus shared an overall nucleotide identity of 85.5–85.6% to human and camel strains of MERS-CoV, with only 64.3–64.6% amino acid identity within the spike gene that is responsible for receptor recognition and attachment [4]. Considering the high diversity of genus-specific bat coronaviruses detected globally [6,27,29], other MERS-related viruses are likely circulating within this host genus.

This study focused on viral discovery within the genus *Neoromicia*, to identify viruses that may be present within this host, as well as detect additional sequences of MERS-related viruses for genetic comparisons. Viral metagenomic sequencing provided an initial assessment of the virome composition from gastrointestinal samples at a host genus-level, detecting sequences from several viral families. However, sequencing outputs produced fragmented coverage of several viruses, making sequence diversity difficult to discern, and lacked suitable sequencing depth as some viruses were only detected from sequencing reads-data. Three viral families (*Coronaviridae*, *Adenoviridae*, and *Herpesviridae*) were selected for further investigation by analysing samples pooled per host species. Conventional nucleic acid detection based on conserved gene regions confirmed diverse sequences of adenoviruses and herpesviruses, as well as the recovery of the complete coding genome of another MERS-related virus.

## Materials and methods

### Ethics statement and sample collection

This research was conducted with the approval of the University of Pretoria Animal Ethics committee (Project no. EC054-14 and EC059-14). Permits were obtained for bat sample collection from the South African provinces involved: the Department of Economic Development, Environment and Tourism Limpopo province directorate- wildlife permit no. CPM006806; Premier of the Province of Gauteng Nature conservation permit no. CPF6 no. 0027/ no.0109; Department of Agriculture, Conservation, Environment and Tourism no. 000039 NW-07; Mpumalanga Tourism and Parks Agency no. MBP5385. As part of a research programme focusing on zoonotic pathogens in small mammals, specimens were collected from four *Neoromicia* species, in five South African provinces between 2007 and 2015 (S1 Table). Bats were measured for morphological identification [21,30], and either released after sampling or taken as voucher specimens and deposited in the small mammal collection at the Ditsong National Museum of Natural History (Pretoria, SA). Further species confirmation was sought for individuals from which coronavirus RNA was detected with sequencing of the mitochondrial cytochrome c oxidase subunit I (COI) gene [31]. All specimen material collected including faecal pellets, as well as rectal and intestinal tissues from necropsies were immediately frozen in liquid nitrogen and stored at -80°C after returning to the laboratory.

### Sample preparation and viral metagenomic enrichment steps

**Fig 1:**
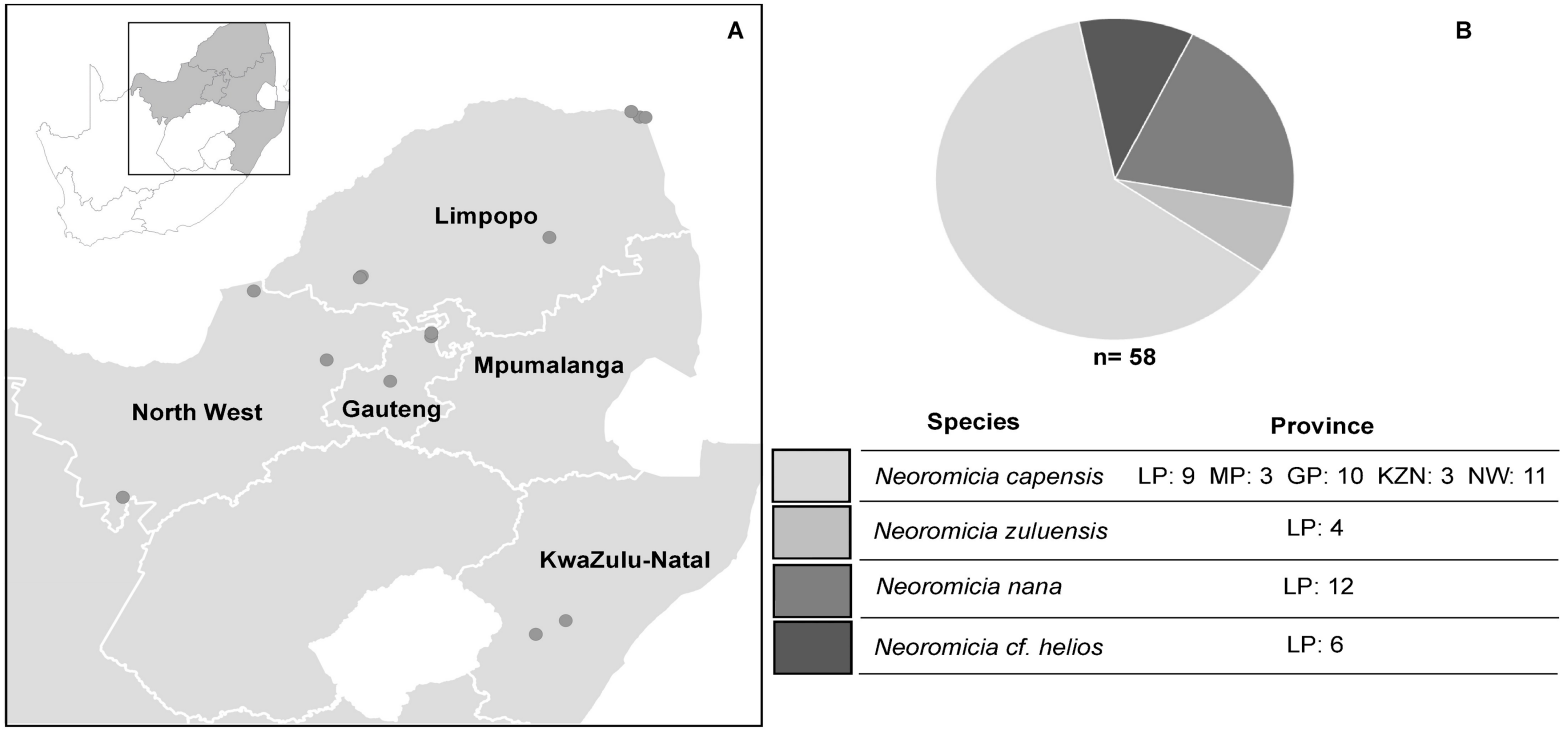
Map of *Neoromicia* sampling locations. **A)** Sampling locations of *Neoromicia* species from 2007 to 2015 in the north eastern regions in South Africa. The map was plotted according to GPS coordinates in QGIS 2.0.1. **B)** Proportion and number of *Neoromicia* species collected per province for the *Neoromicia* virome analysis. LP = Limpopo, MP = Mpumalanga, GP = Gauteng, KZN = KwaZulu-Natal, NW = North West.

**Fig 2:**
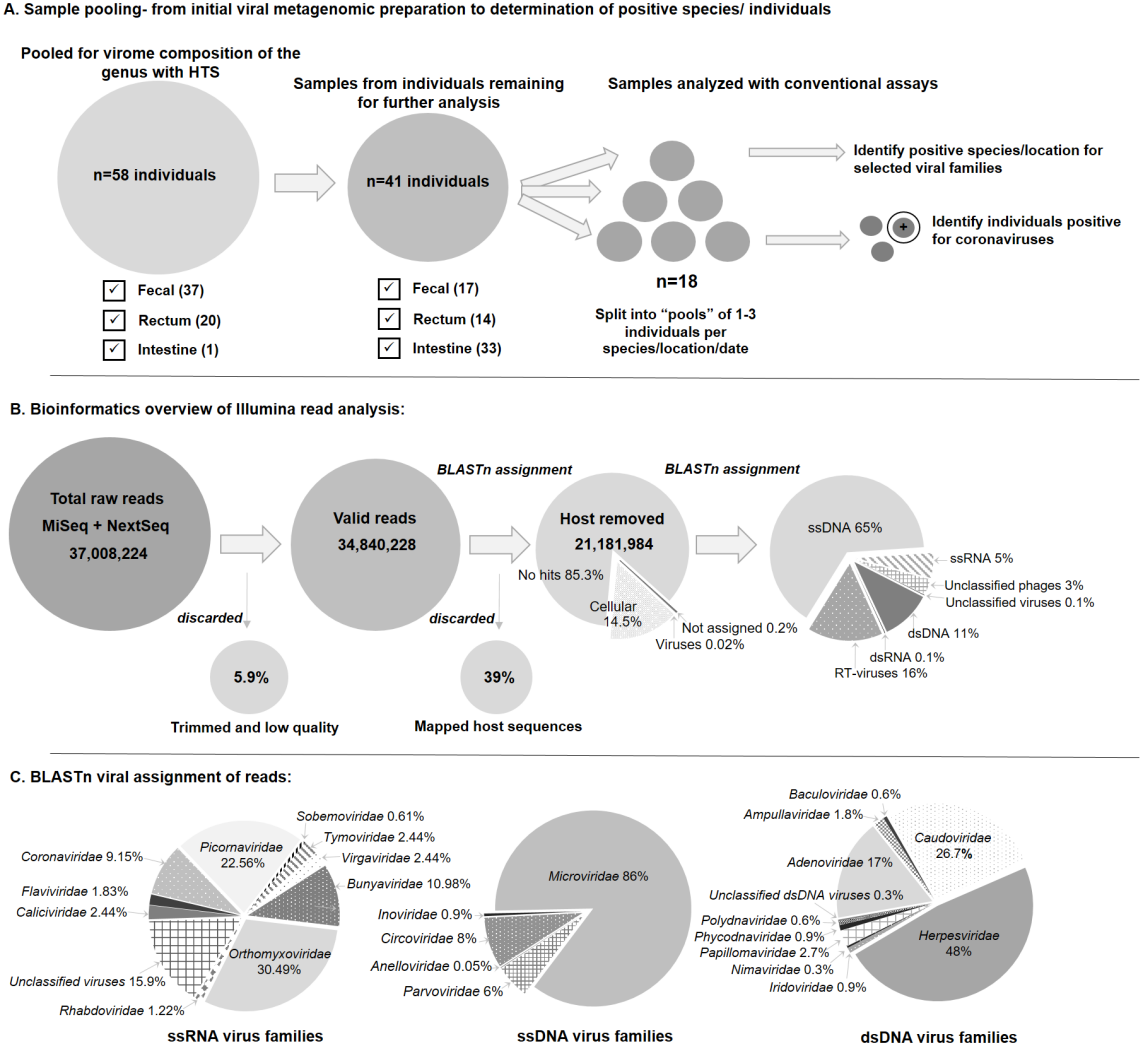
Strategies for sample pooling and data analysis workflows. **A)** The workflow depicts the sample pooling strategies utilized in the study. Faecal and rectal samples were primarily pooled for initial viral metagenomic sample processing. Subsequently, remaining sample material (faecal homogenates, rectum, or intestine) available for individual bats were pooled according to species and location to associate selected viral families with specific species. A further step was implemented to determine specific host individuals harbouring coronaviruses in order to clarify exact sampling locations. **B)** Bioinformatics data analysis of Illumina reads with a developed workflow in CLC genomic workbench that trims data for quality, removes adaptors and depletes host reads based on set mapping parameters. Remaining reads were taxonomically assigned with BLASTn and MEGAN v6. **C)** Shows the BLASTn assignments of reads according to virus genome types: ssRNA, ssDNA and dsDNA viruses.

Samples from 58 individuals of four *Neoromicia* species were collected over an eight-year time span from the north eastern regions of South Africa (Fig 1). The study focused on samples from the lower gastrointestinal tract (faecal, rectal and intestinal samples), to identify viruses present in these tissues or excreted in faecal material (Fig 2A; S1 Table). Due to the size of these bat species and small quantities of faecal material at times available for collection, sample material for certain individuals were limited. All faecal or tissue samples were used sparingly to enable additional analyses. However, priority was given to sample preparation for high throughput sequencing in order to determine the virome composition of the analysed host genus. Therefore, if sample material consisted of very small quantities of faecal or rectal material, these samples were completely used for this step. For high throughput sequencing sample preparation, faecal and rectal samples of all bats were pooled and processed by mechanical homogenization in 400 μl PBS (Lonza) with two stainless-steel beads (Qiagen). A Tissue Lyser II system (Qiagen) was utilized, shaking at 30Hz for 60 seconds. The lysates were cleared by low temperature (4°C) centrifugation for 10 minutes at 10,000x g. Cleared supernatants were pooled and filtered through a 0.45 μM cellulose acetone syringe filter (Corning Incorporated) to remove large particulate matter. The filtrate was ultracentrifuged at 130 000 x g for 2 hours at 4°C. The pellet was resuspended in 200 μl PBS and DNase treated with Turbo DNase (Ambion) before total nucleic acid extraction (ZR Viral DNA/RNA Kit, Zymo Research). Total nucleic acids were eluted in 35 μl nuclease free water (Ambion) and further depleted of ribosomal RNA with the RiboMinus^™^ Eukaryote System v2 (Ambion); nucleic acids were eluted in 12 μl nuclease free water (Ambion).

### Double stranded cDNA preparation for Illumina sequencing

First stand cDNA synthesis and double stranded DNA was prepared as described in Kohl et al. [32] using the K-mer approach (5’ GACCATCTAGCGACCTCCCANNNNNNNN’ 3 and 5’ GACCATCTAGCGACCTCCCA’ 3) described in Victoria et al. [33]. The double stranded cDNA was purified with AmPure XP beads (Agencourt, Beckman Coulter) and analysed for quality with both the Qubit fluorometer (Life technologies, Thermo Scientific) and Agilent 2100 Bioanalyzer (Agilent Technologies). Size selection of 500bp was performed with AmPure XP beads (Agencourt, Beckman Coulter) and the virome library was prepared with the NEB Next Ultra DNA library prep kit (New England Biolabs, Inc). Libraries were normalized to 10pM and 2pM, respectively, and sequenced on the MiSeq and the NextSeq 500 Illumina sequencers for 150bp paired reads at the Los Alamos National Laboratory (New Mexico, USA).

### Bioinformatics and initial taxonomic assignment

Raw Illumina reads were processed in CLC Bio Genomics workbench (Qiagen) using an ‘in house’ developed workflow (S2 Table). After an initial data QC, reads were trimmed for quality and adapter sequences (removing indexes and primers). Host sequences were depleted by strict mapping to an available full genome of a related bat host using default parameters (*Myotis brandtii* assembly no. GCF_00412655.1). The remaining reads were searched for sequence similarity by BLASTn against the NCBI’s nucleotide (nt) database (downloaded July 2015) using an e-value of <10^−5^. Due to limited bioinformatics computational power available, alternative strategies to reduce run time analysis of BLASTx were implemented. A smaller ‘virus-only’ database was obtained from NCBI (downloaded July 2015) and used for BLASTx analyses of reads and contigs. The BLAST xml output files were subsequently exported to the MetaGenome Analyzer (MEGAN v6) [34] for taxonomic assignments, and were further manually investigated. Combinations of assembly approaches were employed including CLC Bio Genomics workbench assembly, and assemblies in Velvet 1.2.10 [35] of all host depleted reads as well assemblies only after family-level identified reads were exported from MEGAN. Kmer sizes of 21, 33 and 55 were used and all contigs from different approaches were combined and curated.

### Conventional nucleic acid detection for specific viral families

In a conserved approach to confirm the high throughput sequence data of specific viral families, primers targeting conserved gene regions were selected from the literature or designed based on obtained sequence data using CLC Bio Genomic Workbench and assessed with Annhyb v4.9. Confirmatory PCRs were performed using sample material remaining after high throughput sequencing. Due to limited sample quantity, original samples of certain individuals were depleted during metagenomic sample preparation. Therefore, remaining samples (faecal homogenates, rectum, or intestine) available from individual bats were pooled according to species, location and sampling date in order to associate selected viral families with specific species (S3 Table). A further step was implemented to analyse original samples of pools found to harbour coronavirus RNA to determine the exact sampling locations and dates of specific host individuals (Fig 2A). Total nucleic acids were extracted in parallel using the Duet RNA/DNA miniprep plus kit (ZymoResearch). A 20 μl cDNA reaction was prepared with 100 ng random primers (IE HPLC Purified, Integrated DNA Technologies) and Superscript III (Invitrogen), followed by incubation for 20 minutes at 37°C in the presence of 2U RNase H (Thermo Fisher Scientific) and inactivated at 65°C for 10 minutes.

In-house *Alpha*- and *Betacoronavirus* specific hemi-nested RT-PCR assays targeting the RNA dependent RNA polymerase (RdRp) gene adapted from Geldenhuys et al. [27] were utilized for *Coronaviridae* confirmatory PCR. In a final reaction volume of 50 μl, 2 μl cDNA template was combined with 1.25U Dream Taq (Thermo Scientific), 1X Dream Taq buffer, 200 μM deoxynucleoside triphosphate (dNTP), 10 pmol of each first round forward and reverse primer (S4 Table) and nuclease free water (Ambion). First round PCR cycling conditions were as follows: 94°C for 1 minute, with 40 cycles of 94°C for 30 s, 42°C for 30 s, 72°C for 1 min; and a final extension at 72°C for 10 minutes. The nested PCR was similarly set up with alterations to the cycling conditions: 94°C for 1 minute, with 35 cycles of 94°C for 30 s, 42°C for 30 s, 72°C for 45 sec; and a final extension at 72°C for 10 minutes. The RdRp gene of detected coronaviruses were extended with appropriate RdRp grouping unit (RGU) primers as described in Drexler et al. [36].

DNA virus confirmatory nucleic acid analysis was performed for the *Adenoviridae* and *Herpesviridae* families as described in Li et al. [37] and Van Devanter et al. [38], respectively. PCR products of appropriate size were excised, purified (Zymoclean^™^ Gel DNA Recovery Kit, Zymo Research) and sequenced on an ABI 3130 sequencer (Applied Biosystems) at Inqaba Biotech (Pretoria, SA).

### MiSeq coronavirus amplicon sequencing

Amplicon sequencing was used to obtain the complete coding regions of the detected MERS-related betacoronavirus from the intestinal material of sample UP5038. Primers were designed from obtained reads, as well as human, camel and *Neoromicia* MERS-related coronavirus reference genome sequences for 11 nested RT-PCR assays (primers available upon request). Amplicons were generated from randomly primed cDNA in PCR reactions using the Phusion high fidelity DNA polymerase (New England Biolabs, Inc). Amplicon sequencing at an estimated 1000x genome coverage was conducted on Illumina’s MiSeq platform after preparation with a Nextera XT library prep kit at the National Institute for Communicable Diseases Sequencing Core Facility (Sandringham, SA). The genome assembly and annotation was performed in CLC Genomics workbench and open reading frames were identified with NCBI’s ORF finder (https://www.ncbi.nlm.nih.gov/orffinder/) as well as the Craig Venter Institute’s VIGOR software [39].

### Phylogenetic analysis and pairwise estimations

Viral sequences for phylogenetic comparisons were downloaded from GenBank (NCBI). Sequence manipulations and alignments were performed with ClustalW in Bioedit [40]. Pairwise sequence similarity estimations were compared in MEGA v7 [41]. Phylogenetic analyses of selected viral contigs and confirmatory PCR sequences were performed with Bayesian phylogenetics using BEAST v. 1.8 [42]. Maximum clade credibility trees were constructed using suggested models selected from jModelTest.org [43]. Unless otherwise stated, Bayesian MCMC chains were set to 10,000,000 states, sampling every 1000 steps. Final trees were calculated from the 9000 generated trees after discarding the first 10% as burn-in. Computationally expensive analyses were run with the aid of CIPRES [44]. Trees were viewed and edited in Figtree v1.4.2.

### Construction of an overlapping distribution map of known hosts of MERS virus and MERS-related coronaviruses

The African geographical distributions of dromedary camels (*Camelus dromedarius*) and the Cape serotine bats (*Neoromicia capensis*) were plotted in ArcMap v.10.4.1 to indicate possible regions of overlap that may be used as focus areas for coronavirus surveillance. The natural distribution range of dromedary camels was traced from the warm desert, semi-desert, woodland, scrub grassland vegetation subclass [45] based on the range described by Faye [46]. To identify introductions to areas outside the natural range, a Google search (terms included ‘camel rides’ and ‘country’) and Wilson [47] were employed. To create an estimated species distribution model for *N*. *capensis*, the museum records with point localities was extracted from the African Chiropteran Report [21] and modelled using climatic variables from present WorldClim [48], using MaxEnt version 3.3.3k [49]. MERS antibody seroprevalence data for dromedary camels was obtained from available publications as well as hosts from which MERS-CoV and MERS-related viral RNA have been detected [50–55].

### Data submission and accession numbers

The raw sequence data was submitted to the Sequence Read Archive of the NCBI under accession numbers SRR5889194 and SRR58891929. Viral sequences were submitted under accession numbers MF579865-MF579871 and MF593268-MF593281.

## Results and discussion

The study investigated the gastrointestinal virome of the bat genus *Neoromicia*, as viruses excreted in fecal material or present in these tissues (rectum and intestine) pose a greater risk of coming into contact with other animals. *Neoromicia* species do not roost in caves where they can be readily caught and sampled [21]. Instead, most individuals were caught in flight away from their roosts, and therefore fewer individuals were available to investigate. The samples utilized in this study were processed with methods similar to those that have proven to be successful in bat virome analyses–with enrichment methods for conserving viral particles and reducing sequencing of host and non-viral nucleic acids [11,15,17,56]. Furthermore, an additional host ribosomal RNA depletion step was incorporated to simplify bioinformatics analysis. Prepared Illumina libraries were sequenced on the MiSeq platform as an initial examination, followed by sequencing on the NextSeq500. Combined data outputs produced 37 million reads of which 34.8 million remained after quality and adaptor trimming with an in-house developed workflow in CLC Genomics Workbench (Fig 2B). As no *Neoromicia* host genomes are available for sequence removal, the genome of *Myotis brandtii*, another species within the family Vespertilionidae, was utilized. Despite enrichment efforts, application of strict mapping parameters within the CLC workflow removed nearly 40% of reads as host (Fig 2B).

Before assembly into contigs, reads data were analysed for sequence similarity by BLASTn against the nucleotide database of the National Center for Biotechnology Information (NCBI). Of the remaining 21.2 million reads, 14% were attributed to cellular organisms (host and non-viral microbial reads), with less than 1% designated as viral in origin; which is comparable to other virome studies [15,18]. As with most metagenomic studies performed to date, the largest portion of reads (85%) were assigned as ‘non-hits’ by BLASTn analyses, as they did not match any available sequence data in the NCBI database [11,56].

Reads were assembled into contigs using CLC Genomics Workbench and Velvet assembler, and combined for the best results; often producing overlapping contigs. Depending on specific inputs (e.g. varying kmer lengths), the number of contigs ranged from 222,343 to 566,951. Superior contig lengths were more frequently found from CLC assemblies than those from multiple Velvet configurations with different kmer lengths. Again, less than 1% of all contigs were assigned as viral with BLASTn analyses. Contigs and unassembled reads assigned to specific virus families from BLAST outputs were inspected manually before being considered as valid (Fig 2C). Many of the assignments were deemed as false positives due to poor e-values, low complexity sequences (such as repeat runs of nucleotides) or having greater similarity matches to host/mammalian sequences [57]. Reads and contigs (e.g rhabdoviruses, orthomyxoviruses and caliciviruses) identified as such were discarded from further analyses (Fig 2C). Despite a low percentage of viral reads, sequence data from eight mammalian-infecting virus families were detected, including the *Adenoviridae*, *Circoviridae*, *Coronaviridae*, *Herpesviridae*, *Parvoviridae*, *Papillomaviridae*, *Picornaviridae* and *Phenuiviridae*. In agreement with other virome investigations, many of detected sequences shared low genetic similarities to known viruses due to a lack of available sequence diversity for comparison [11,14]. This lack of available sequence data may only be overcome by further reports documenting the viral diversity of bats as well as other hosts. Though non-mammalian infecting viruses (phages and insect viruses) were identified, they were outside the scope of the study and not investigated any further.

### New viral species in the family *Circoviridae*

Viruses from the family *Circoviridae* have small, single-stranded, DNA genomes (1.8–3.8 kb). Members of the genera *Circovirus* and *Cyclovirus* have proven to be readily detectable with metagenomic approaches [58,59]. A large diversity of these viruses has been reported from viromes of various mammalian hosts, including both domestic and wildlife species [58,59]. Infections of these viruses in domestic animals have been known to cause significant annual economic losses to the poultry and pork industries [58]. The risk of zoonotic transmission for members of the *Circoviridae* is still largely unknown.

**Fig 3:**
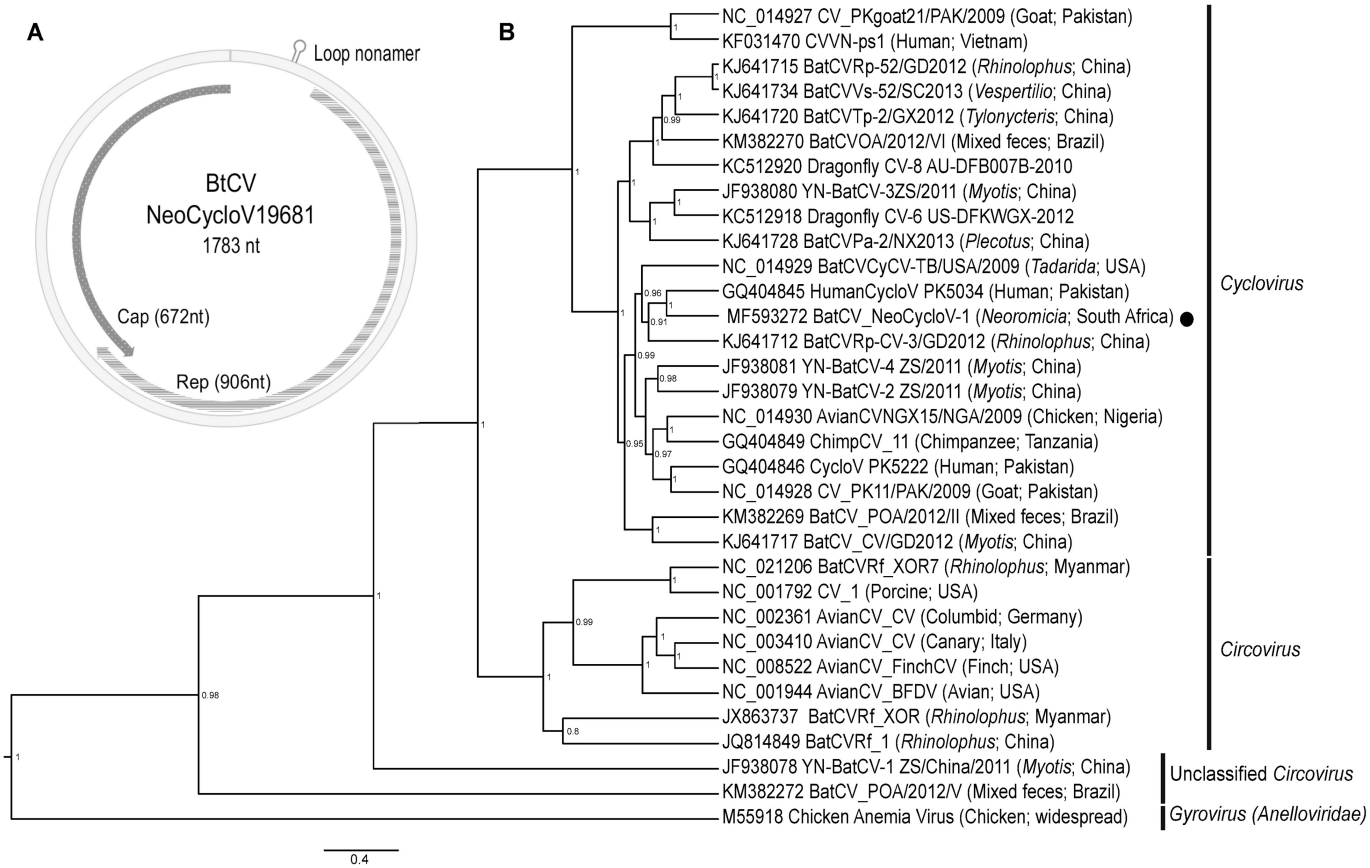
Novel *Neoromicia* cyclovirus. **A)** The circular genome organization of NeoCycloV-1 (19681/RSA) is shown with the rep and cap genes in opposing directions. The characteristic *Cyclovirus* nonamer is indicated at nucleotide position 99. **B)** Bayesian phylogeny of representative species of the *Circoviridae* using complete genomes. The phylogenetic tree was constructed in BEAST v1.8 using GTR plus invariant sites and gamma distribution substitution model. The genera are indicated on the right side of the sequence names (with indicated GenBank accession numbers), and the *Neoromicia* cyclovirus from this study is indicated with the black circle. Chicken anemia virus from the *Anelloviridae* family was used as an outgroup. Posterior probability values of less than 50% were omitted.

A putative novel *Cyclovirus* species was identified from the collective *Neoromicia* virome. The full-length genome of 1783bp was characterized to encode a 906bp Rep (replicase-associated protein) gene and 672bp Cap (capsid protein) gene (Fig 3A). The full genome phylogeny of the novel virus named *Neoromicia-*associated cyclovirus 1 strain 19681/RSA (or NeoCycloV-1), shows grouping within the genus *Cyclovirus*; including bat cycloviruses from the USA and China, as well as cycloviruses from human stool originating in Pakistan (Fig 3B) [11,14,58]. As with other cycloviruses, the genome of NeoCycloV-1 is smaller (1.7kb) than in relation to circoviruses (typically 2kb). Furthermore, characteristic to cycloviruses is the rolling circle replication initiation 5’ intergenic loop nonamer (5’TAATACTAT’3) identified at position 99-107bp of the genome (Fig 3A), as well as the absent 3’ intergenic regions between the two major open reading frames [58].

The International Committee on Virus Taxonomy (ICTV) requires sequence similarities of less than 75% overall genome identity and less than 70% amino acid similarity of capsid proteins for designation as different species [58,59]. NeoCycloV-1 shares overall genome nucleotide identities of 44.2–64.9% between compared cycloviruses, with less than 40.8% identity to members of the genus *Circovirus* (S5 Table). The capsid protein shared amino acid similarities of only 20.6–49.1% to all compared viruses in the family. In agreement with additional species demarcations suggested by Li et al. [58], requiring at least 85% amino acid similarity within the Rep protein, the NeoCycloV-1 shares between 48.4–73.3% similarity to compared cycloviruses. Similarities of the NeoCycloV-1 are well within accepted common thresholds for species in the genus *Cyclovirus*. Given it meets the ICTV criteria for species demarcation, we propose the new species, *Neoromicia*-associated cyclovirus 1 (strain 19681/RSA) within the genus *Cyclovirus*. Interestingly, the closest relative of the putative new *Neoromicia* cyclovirus originated from human stool; with an overall nucleotide identity of 64.9%, and amino acid similarities of 73.3% and 49.1% for the Rep protein and capsid protein, respectively. Additional cycloviruses are likely be detected in bat species from Africa, which may be more closely related.

### Contigs identified from the *Neoromicia* virome

Various contigs were identified from the data analysis of the *Neoromicia* virome. Shorter, fragmented contigs were obtained from the *Phenuiviridae* family as well as the *Parvoviridae* and *Papillomaviridae* (the latter two families are detailed in S1 File). The longest length contigs (1077-1806bp) originated from the *Picornaviridae* and *Adenoviridae* families.

#### Phenuiviridae

Recent taxonomic changes have reported the elevation of the family *Bunyaviridae* to the order *Bunyavirales*, with the known genera being reassigned as families (ICTV *Bunyavirales* taxon change, code 2016.030a-vM). Some families comprise arthropod-borne viruses, including a number of important public health pathogens such as severe fever with thrombocytopenia syndrome virus (SFTSV). Despite several contigs assigned as belonging to members of the *Bunyavirales*, only one was considered a valid viral contig after further inspection (as other contigs were shown to be false positives attributed to mammalian/host sequences).

**Fig 4:**
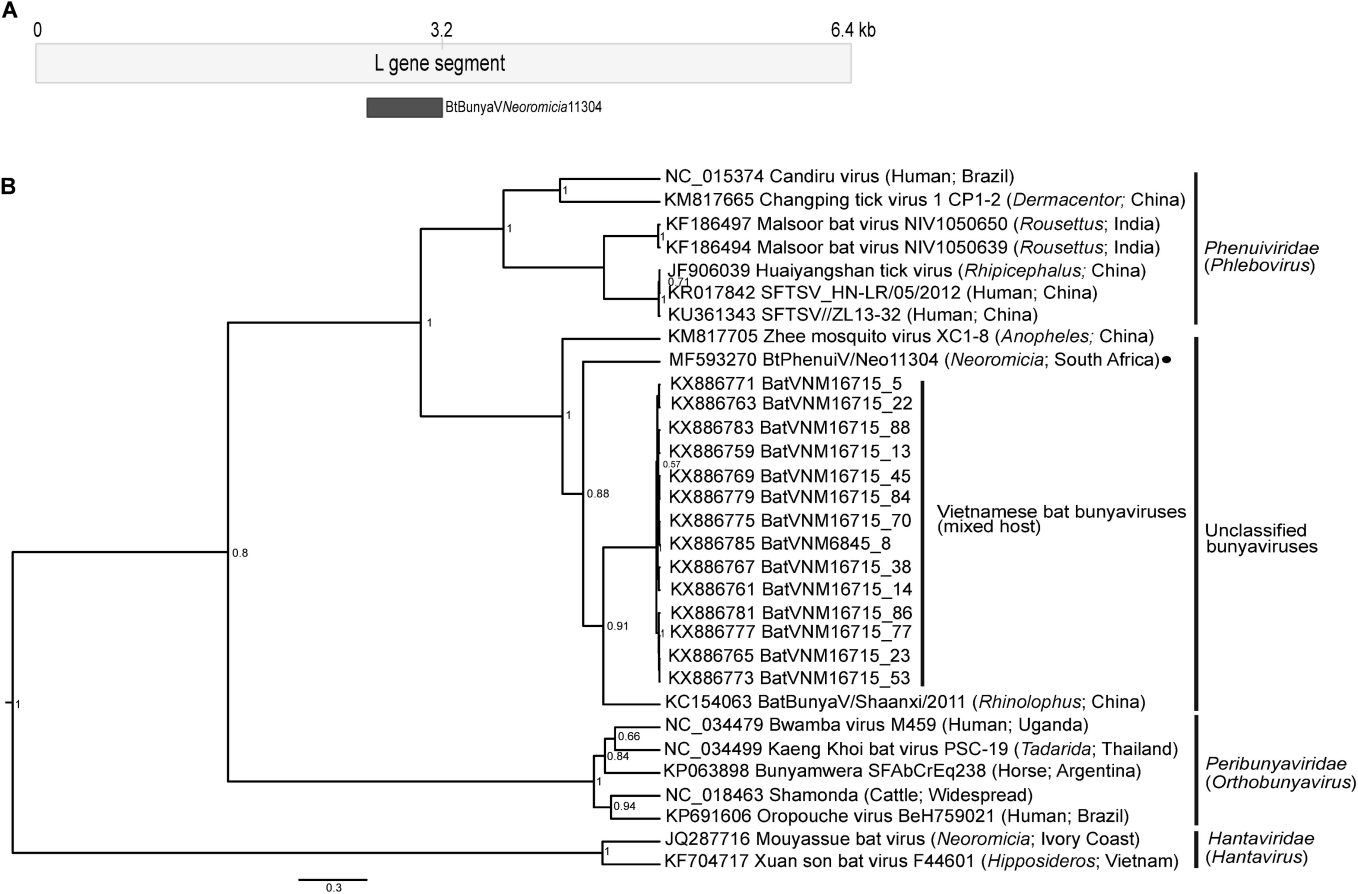
*Phenuiviridae* sequence identified from the *Neoromicia* virome. **A)** Alignment of the *Phenuiviridae* contig in reference to a typical L segment gene. **B)** The phylogenetic tree was constructed with a 267 bp region of the bunyavirus L genome segment using BEAST v1.8 with the GTR substitution model plus invariant sites. Relevant genera are shown on the right and GenBank accession numbers of each sequence are provided; the novel *Neoromicia* bunyavirus sequence from this study is indicated with a black circle. SFTSV = Severe fever with thrombocytopenia syndrome virus.

The contig, Bt/PhenuiV/Neo11304/RSA, of 267bp aligned to a region of the L gene segment in the RNA dependent RNA polymerase (RdRp) region that may typically be used for phylogenetic analysis. Its closest matches were to novel Asian bunyaviruses identified in China from bats in the genus *Rhinolophus*, as well as mixed species bat guano from Vietnam (Fig 4) [8,60]. These viruses were classified as unassigned, though considered part of the *Peribunyaviridae* family, and shared between 63.9–68.7% pairwise nucleotide identities to the contig Bt/PhenuiV/Neo11304/RSA (S6 Table). A mosquito virus identified in China shared a basal position to the lineage of these bat viruses (60–61.8%). As in this study, these bat-associated virus sequences were identified from gastrointestinal specimens (faecal, rectum or gastrointestinal swabs), and therefore the possibility that these viruses may have originated from arthropods consumed by these insectivorous bat hosts cannot be excluded. Alternatively, part of the replication cycle of these bat-associated bunyaviruses may be shared in arthropods hosts.

Bt/PhenuiV/Neo11304/RSA shares low homologies (38.2–42.2% nucleotide identities) to viruses in the family *Peribunyaviridae* (such as Kaeng Khoi bat virus or Oropouche virus from the genus *Orthobunyavirus*). Similarities to members of the genus *Phlebovirus*, in the family *Phenuiviridae*, which includes SFTSV and Malsoor *Rousettus aegyptiacus* bat virus, range between 45–49% [61]. As a comparison, the *Neoromicia* hantavirus (*Hantaviridae*), Mouyassué virus from the Ivory Coast, shares only 32.9% nucleotide identity to the contig detected in this study [25].

The phylogeny indicates that Bt/PhenuiV/Neo11304/RSA and other bat viruses from China and Vietnam may be more related to the family *Phenuiviridae* (known to be frequently transmitted by arthropods) than to the family *Peribunyaviridae*. Though this is only tentatively based on a short segment of the RdRp gene and would require confirmation with complete genome segments. This short sequence provides an initial indication that members of this family are present in the genus *Neoromicia* and constitutes the only sequence data of a virus possibly associated with the *Phenuiviridae* family from insectivorous bats in Southern Africa. The low homology of Bt/PhenuiV/Neo11304/RSA to other available viruses from the *Bunyavirales* order is likely attributable to lacking comparable sequence data. Additionally, further viral surveillance activities directed toward insectivorous bats would greatly benefit from comparative surveillance initiatives of the arthropods frequently consumed by the bats.

#### Picornaviridae

Picornaviruses are small, single-stranded, positive sense RNA viruses with approximately 7.1–8.9 kb genomes [62]. These viruses are diverse and exploit several routes of transmission; resulting in respiratory disease caused by rhinoviruses, gastroenteric infections caused by entero- and kobuviruses, as well as hepatic infections caused by hepatitis A virus [62]. Due to rare reports of zoonotic spillover, picornaviruses have not generally been considered as zoonotic. The zoonotic potential of the few documented bat picornaviruses are thus unknown [11,62–64]. Bat-associated picornaviruses have been reported from Asia, Europe, Africa and the USA, and some have necessitated the creation of additional genera (e.g. the genus *Mischivirus*) in a family that already contained 29 officially recognized genera [11,63]. Other bat-associated picornaviruses have also grouped with the genera *Sapelovirus*, *Kobuvirus* and *Enterovirus* [11,14,62,64].

**Fig 5:**
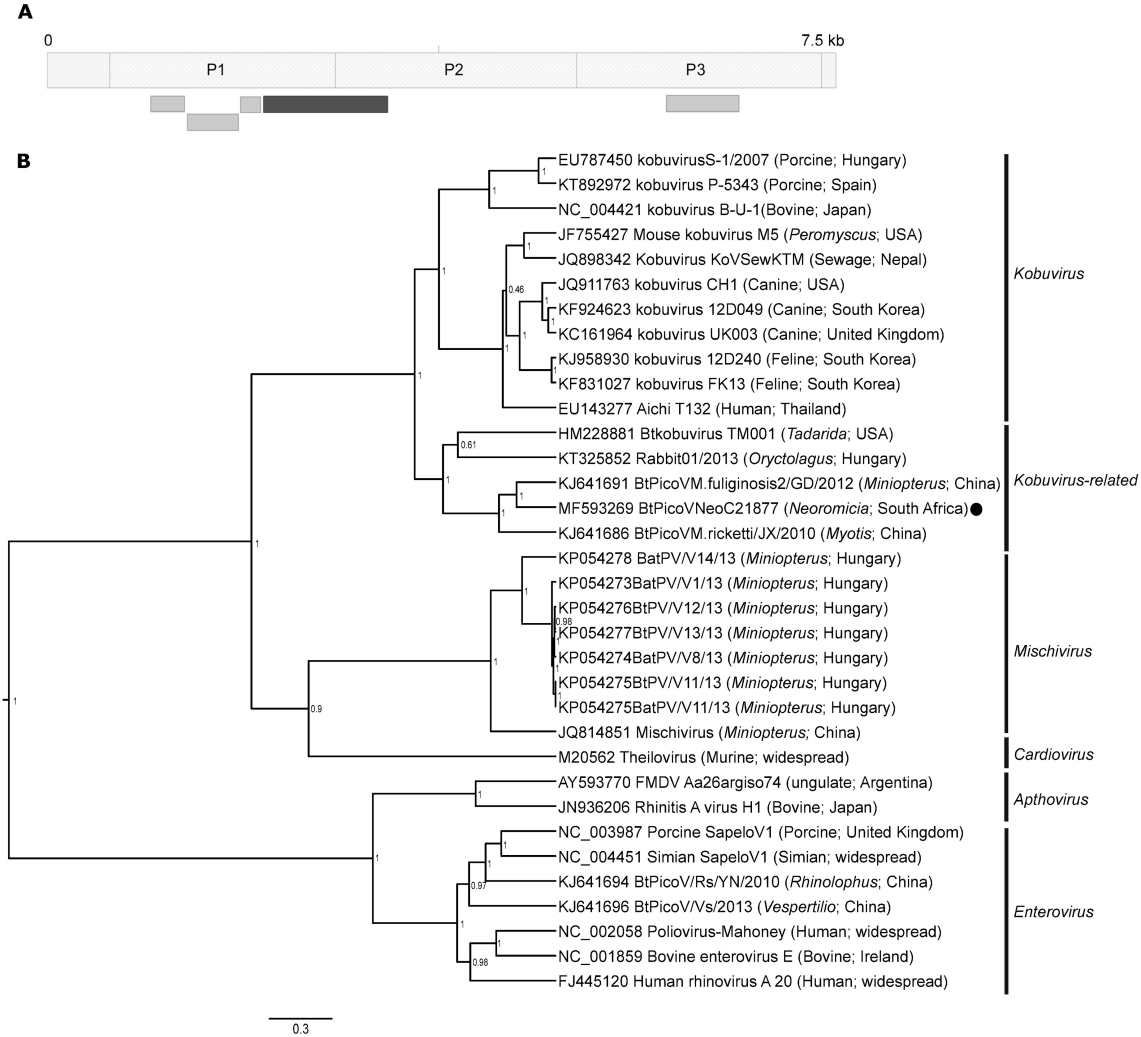
Novel *Neoromicia* picornavirus. **A)** Overview of the confirmed picornavirus contig alignment positions in reference to a typical picornavirus genome. The dark grey contig (1077bp) of the P1/P2 region was used to construct a Bayesian phylogeny. **B)** The maximum clade credibility tree constructed in BEAST v1.8 used the GTR plus invariant sites and gamma distribution substitution model. The genera are indicated on the right side of the sequence names (with GenBank accession numbers); the *Neoromicia* picornavirus from this study is indicated with a black circle. Posterior probability values of less than 50% were omitted.

Several contigs from the *Picornavirales* order were identified from the metagenomic data, some constituting insect infecting iflaviruses or were deemed as low complexity sequences upon further curation of the sequences (Fig 5A). A number of the contigs of mammalian-infecting picornavirus origin could be consolidated into a 1077bp contig, aligning to the P1 and P2 genome regions that encode for structural proteins (Fig 5B). Phylogenetic analysis groups the novel *Neoromicia* picornavirus sequence (BatPicV/NeoC21877/RSA) in a sister clade to those belonging to the genus *Kobuvirus* (Fig 5B). Pairwise alignments to other unassigned bat picornaviruses as well as a novel rabbit picornavirus within the clade indicate that they share between 55.1–73.6% nucleotide identity and 48–76.7% amino acid similarity. The *Miniopterus fuliginosus* bat picornavirus (BtMf-PicoV-2/GD2012) identified in China was the closest available relative [11]. The rabbit picornavirus had been tentatively placed into the *Kobuvirus* genus as it possessed certain qualifying characteristics [65]. However, this novel clade shares much lower similarities (46.3–54.9% nucleotide identities and 38.9–47.4% amino acid similarities) to other classified kobuviruses and decreased to below 39.7% nucleotide identity when compared to the genera *Mischivirus*, *Enterovirus*, *Sapelovirus*, *Apthovirus* and *Cardiovirus*.

To our knowledge, this is the first indication of a bat picornavirus from an insectivorous bat genus in Africa. The virome analysis of *Eidolon helvum*, a fruit bat from Ghana, also identified sequences of kobuvirus-related picornaviruses from urine; though it cannot be directly compared due to non-overlapping regions [17]. Kobuvirus species have been documented to infect both humans and domestic species such as cattle, dogs and cats, causing severe gastroenteritis [65]. Future surveillance for bat-associated kobuviruses will enable determination of zoonotic potential and discern whether these viruses may be classified as a new genus.

### Viral families detected with insufficient sequencing depth

**Fig 6:**
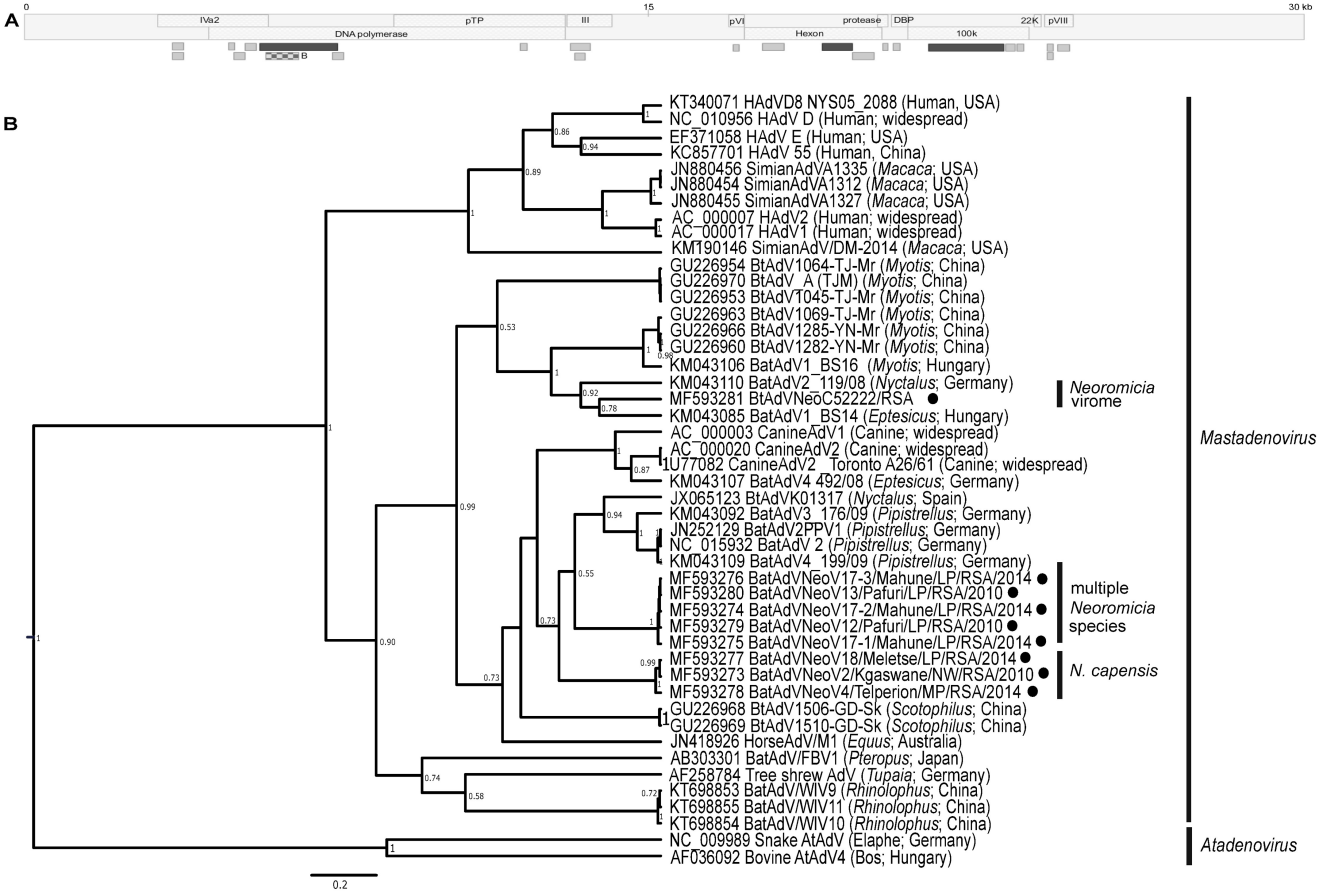
*Neoromicia* adenovirus sequences. **A)** Overview of confirmed mastadenovirus contigs from the *Neoromicia* virome created with Velvet and CLC assemblers as they align to a characteristic mastadenovirus genome. The dark grey contig was used in B, along with the amplicons produced by conventional PCR (depicted by the checkered block). **B)** Bayesian phylogenetic tree of a 237 bp region of the DNA polymerase gene. The phylogeny was constructed in BEAST v1.8 using the Hasegawa, Kishino and Yano (HKY) substitution model plus gamma distribution model suggested by J-model test. The MCMC chain was set to 20,000,000 generations sampled every 2000 steps, with a 10% burn-in of the first generated trees. Adenovirus sequences detected from this study are shown with black circles, and bat species from which adenoviruses originated are indicated on the right side of the sequence names. Posterior probability values of less than 50% were omitted. GenBank accession numbers are shown next to sequences.

Low sensitivity and insufficient depth of sequencing was observed as fragmented viral genome coverage, and further noted from discrepancies between BLASTn assignments of reads and that of contigs; as reads data suggested the presence of more viral families than contigs. For example, no contigs were produced from the *Coronaviridae* family, though several pairs of reads were detected across distant genome regions. Several contigs of <430bp were designated as herpesvirus sequences, though were discarded after manual curation as being only mammalian-derived sequences. Some contigs were still identified as herpesvirus, though had poor e-value support. Severely fragmented viral genome coverage was observed for the *Adenoviridae*, with contigs aligning to multiple gene regions ranging in length from 200 to 1826bp (Fig 6A). Estimation of adenovirus genome coverage is further complicated by the detection of divergent sequences that align to diverse adenovirus reference genomes. These limitations may be attributed to overall insufficient depth of sequencing, the bioinformatics analyses methods implemented (BLASTn), or biases incorporated by certain nucleic acid preparation techniques for library construction—such as the use of randomly primed cDNA [66,67]. Similar events have been noted in the literature; where viruses were unsuccessfully or partially sequenced by high throughout sequencing though readily amplified with conventional PCR assays [10,68]. Due to limited computational resources, and surveillance emphasis focusing on identification of mammalian viral families with zoonotic potential, BLASTn analyses was deemed sufficient. However, more divergent viral sequences, particularly for non-frequently sequenced genome regions, may have been better detected with a BLASTx analyses using the complete NCBI nr database.

To confirm the presence of the herpes- and coronavirus sequences as well as determine the sequence diversity of adenoviruses, these three viral families were selected for further investigation with conventional nucleic acid detection assays based on conserved gene regions [37,38]. Analysis was conducted on sample material remaining from individuals following sample preparation for Illumina sequencing. Due to limited quantities of sample material, original samples of certain individuals were depleted during metagenomic sample processing (Fig 2A). Therefore, as selected confirmatory nucleic acid tests could not be performed for each individual bat, available samples (faecal homogenates, rectal or intestinal samples) were pooled according to species, sampling date and location to associate selected viral families with specific species (S3 Table). Original sample material from pooled samples found to harbour coronavirus RNA were separately assayed to trace back the exact sampling location and date (Fig 2A).

#### Adenoviridae

Adenoviruses are double-stranded DNA viruses of approximately 35kb, which may cause a variety of respiratory and gastrointestinal infections within their human, domestic pet animal, and wildlife hosts [37]. Surveillance initiatives in multiple countries have identified bats as important hosts for the genetic diversity and evolutionary history of adenoviruses [19,69,70]. Adenoviruses have been shown to be capable of interspecies transmission and establishment within a new host due to the close relatedness of certain bat and canine adenoviruses [19]. Even though bat adenoviruses could potentially be transmitted via the aerosolization of bat faecal material, their potential zoonotic risk to humans seems low [71].

The PCR assay selected to confirm *Neoromicia* adenovirus sequence diversity was based on a conserved region (260bp) of the DNA polymerase gene [37]. This region overlapped with a 860bp contig (BtAdV/NeoC52222/RSA) identified from the high throughput sequencing data, though few bat adenovirus sequences of greater than 400bp were available for comparison. PCR analysis identified adenovirus DNA in eight of the remaining pooled samples. These sequences represented three clades (Fig 6B). The *N*. *capensis* sequences originating from three different provinces, and sampled over five years, grouped together and shared between 97.7–99.6% nucleotide identities. This clade was 78.7–79.8% identical to sequences in the mixed species clade of *N*. *nana*, *N*. *zuluensis* and *N*. *cf*. *helios* hosts from Limpopo province, which shared 98.8–100% nucleotide identity. Two of these sequences (BtAdV/NeoV13/LP/RSA/2010 and BtAdV/NeoV17-3/LP/RSA/2014) were identical, even though they were identified from bats collected four years apart in different locations of the Limpopo province. The polymerase contig BtAdV/NeoC52222/RSA, identified directly from the *Neoromicia* virome, shared lower similarities (64.7–65.5%) to the other groups of *Neoromicia* adenovirus sequences.

When compared to non-*Neoromicia* adenovirus sequences, contig BtAdVNeoC52222 shared the greatest similarity (79%) to a vespertilionid adenovirus from *Eptesicus serotinus* from Hungary [70]. The other *Neoromicia* adenovirus sequences shared greater nucleotide similarities (72.2–79.9%) to bat adenovirus B (BtAdV2) from *Pipistrellus pipistrellus* in Germany and other vespertilionid species from Hungary [70,72]. The DNA polymerase assay did not amplify any sequences able to confirm the origin of contig BtAdVNeoC52222—detected directly from the *Neoromicia* virome. The contig may have originated from one of the individuals for which specimen material was depleted, and was thus unable to be further analysed with the nucleic acid detection assay. The metagenomic analysis approach was unable to identify the diversity of adenovirus sequences confirmed with a conventional PCR approach–highlighting a limitation of the methods utilized for sample preparation or a lack of sufficient depth of sequencing.

#### Herpesviridae

The *Herpesviridae* is a large family of viruses subdivided into three subfamilies, the *Alphaherpesvirinae*, *Betaherpesvirinae*, and *Gammaherpesvirinae*. Bat-associated herpesviruses have been detected from all three subfamilies, with several that have yet to be classified [73,74]. Herpesviruses are known to cause recurrent or latent infections that may later become reactivated within particular cell types. Though no bat herpesviruses have been linked to zoonotic infections, a gammaherpesvirus isolated from a *Myotis* cell line was shown capable of growth in human derived cell lines [73]. Evolutionary analysis of gammaherpesviruses originating from a number of host orders suggest that multiple host switching events and independent viral cospeciation of lineages may previously have occurred; with bats and primates acting a sources of genetic diversity [75].

**Fig 7:**
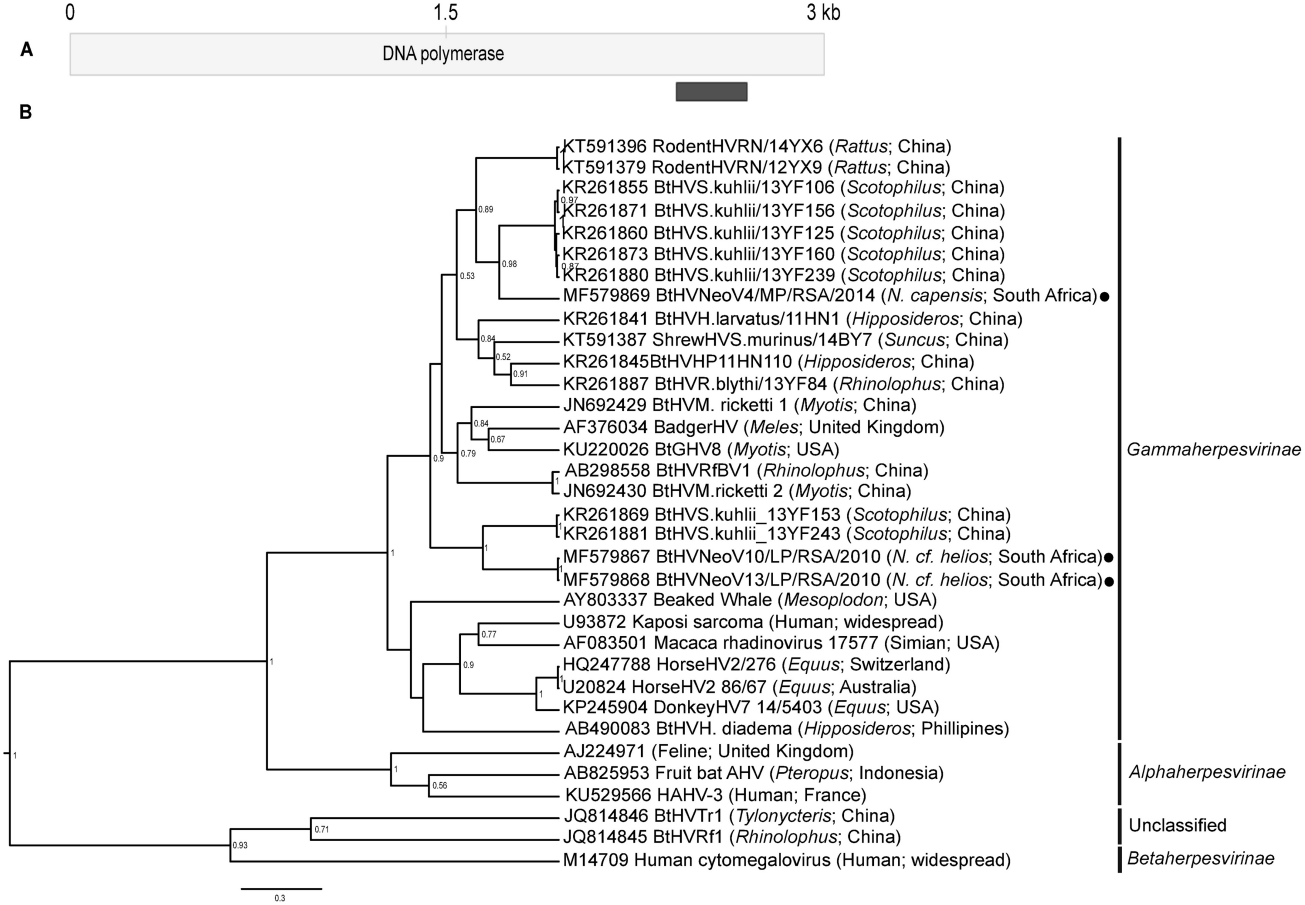
*Neoromicia* herpesvirus sequences. **A)** Region of the herpesvirus DNA polymerase targeted with the molecular detection assay [38]. **B)** The phylogenetic tree of a conserved 215 bp segment the herpesvirus DNA polymerase gene. The phylogeny was constructed in BEAST v1.8 using the GTR plus invariant sites and gamma distribution substitution model as suggested by J-model test. The *Neoromicia* herpesvirus sequences from this study are indicated with the black circles, and the species from which they originated are shown. The subfamily that compared sequences may possibly belong to, are indicated with GenBank accession numbers on the right side of the phylogenetic tree.

The conventional nested PCR assay used for herpesvirus detection also targeted a conserved region of the DNA polymerase gene [38]. The assay confirmed the presence of three herpesvirus sequences from *N*. *cf*. *helios* and *N*. *capensi*s pooled tissue from the Limpopo and Mpumalanga provinces, respectively. The two herpesvirus sequences from *N*. *cf*. *helios* shared 99.5% nucleotide identity, and only 60.9–61.4% nucleotide identity to the sequence from *N*. *capensis*. These DNA polymerase sequences were compared to Genbank sequences and grouped with other known bat gammaherpesviruses–particularly *Scotophilus kuhlii* bat herpesviruses from China (Fig 7). The *N*. *cf*. *helios* herpesvirus sequences were more divergent (44.7–67.4% nucleotide similarity) in comparison to other bat herpesviruses than the sequence from *N*. *capensis*, which shared 74.9% similarity to BtHVSK_13YF125 (KR261860) [74]. The lack of larger sequenced regions of African bat herpesviruses from the subfamily may have contributed to the unsuccessful recovery of *Neoromicia* herpesvirus sequence information from the metagenomic data analysis, which would have improved phylogenetic placement.

#### Coronaviridae

Coronaviruses have large positive sense single-stranded RNA genomes of approximately 30kb. The *Coronavirinae* subfamily is split into four genera; species from the genera *Alphacoronavirus* and *Betacoronavirus* mainly infect mammalian hosts, while the species from the genera *Gammacoronavirus* and *Deltacoronavirus* largely infect avian hosts (with sporadic detections in mammals) [6]. The genus *Betacoronavirus* is divided into four lineages (A-D), with lineage C referring to MERS-CoV and related coronaviruses [76]. A number of paired reads suggested the presence of both alpha- and betacoronaviruses, with several pairs sharing high similarity to the betacoronavirus NeoCoV/PML-PHE1 [4]. Conventional nucleic acid detection with a genus-specific nested RT-PCR assay amplifying a 268-440bp conserved region of the RdRp gene confirmed coronavirus RNA in available specimen material from two *N*. *capensis* individuals. Sequences were extended to 648bp with combinations of RdRp grouping unit (RGU) primers [36].

**Fig 8:**
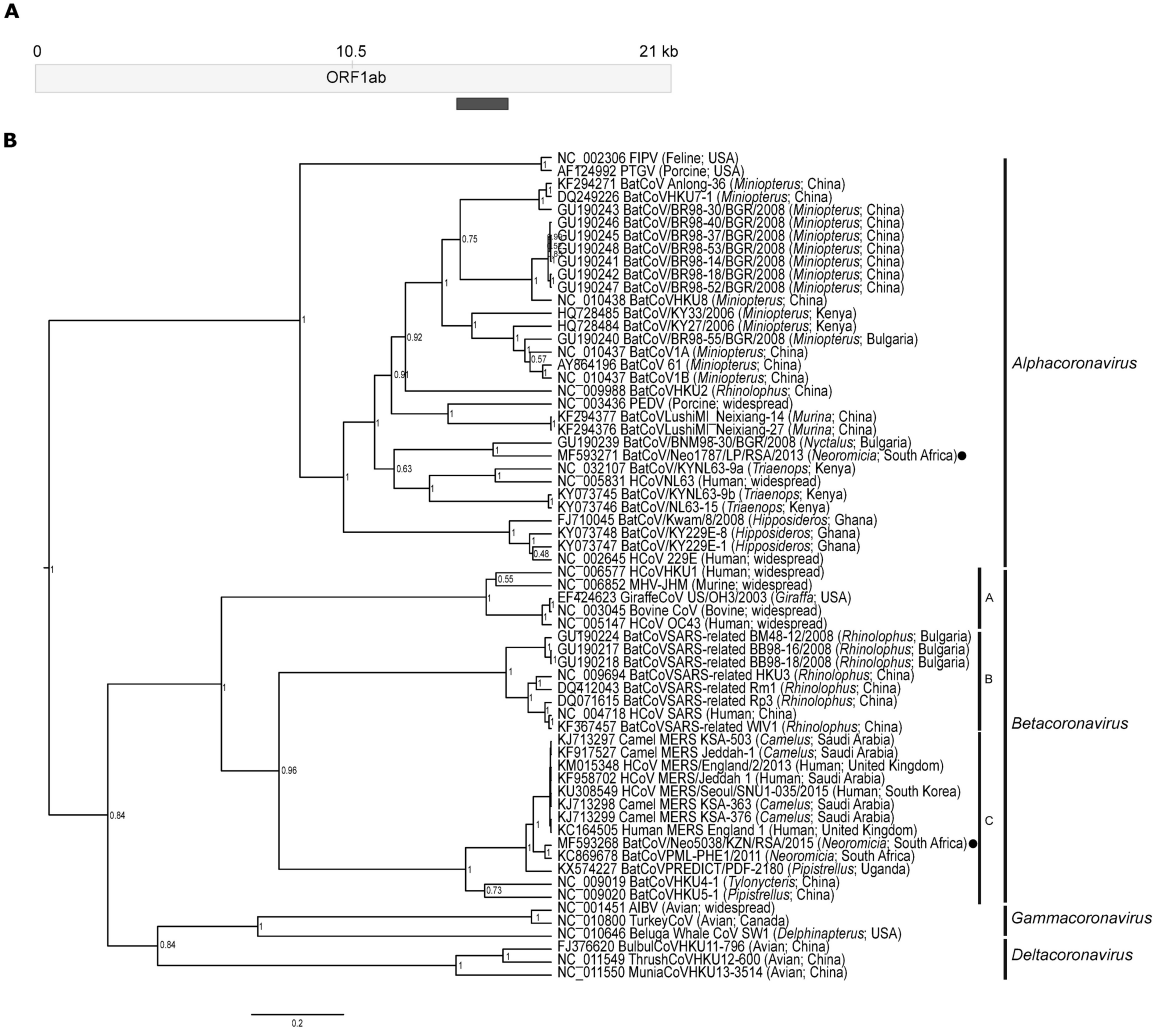
Conserved sequences of identified *Neoromicia* coronaviruses. **A)** Region of the coronavirus ORF1ab (within the RdRp gene) targeted with the molecular detection assay. **B)** The coronavirus phylogenetic tree generated using the 605bp region was constructed in BEAST v1.8 using the GTR plus invariant sites and gamma distribution substitution model. The MCMC chain was set to 20,000,000 generations, sampled every 2000 steps, with a 10% burn-in of the first generated trees. Coronavirus genera and *Betacoronavirus* lineages are indicated on the right side of the sequences. GenBank accession numbers are shown with each sequence and coronaviruses detected in this study are indicated with a black circle. Posterior probability values of less than 50% were omitted.

The alphacoronavirus BtCoV*Neoromicia*1787/LP/RSA/2013 from intestinal tissue of an *N*. *capensis* bat, shared high similarity (94.6–95.3% nucleotide identities) to the previously mentioned South African alphacoronaviruses reported from the same host genus [27,28]. The host species was confirmed by morphological identification and cytochrome C barcode analysis [21,31]. The *Neoromicia* alphacoronaviruses form an independent lineage with genetically similar members detected throughout the geographic distribution of the *N*. *capensis* host species [27,28]. BtCoV*Neoromicia*1787/LP/RSA/2013 was utilized as a lineage representative for these *Neoromicia* alphacoronaviruses in Fig 8 (as the other sequences were of an insufficient length for comparison). The closest relatives to this lineage was a *Nyctalus lysleri* bat alphacoronavirus (BNM98-30/BGR/2008) from Bulgaria, as well as a clade containing the human coronavirus NL63 (HCoVNL63) and related *Triaenops* bat viruses from Kenya [36,77,78]. The *Neoromicia* and *Nyctalus* alphacoronaviruses shared between 75.5–79.2% nucleotide identities to HCoVNL63, which is somewhat lower than the similarities of the NL63-related *Triaenops* bat viruses within this gene region (76–84% nucleotide identity) (S7 Table). Comparatively, *Hipposideros* bat coronaviruses identified in Ghana were suggested to have been possible early progenitors of the human alphacoronavirus HCoV229E [79], and shared greater similarities of 87–92% nucleotide identity to HCoV229E. Further surveillance and sequence analysis of the alphacoronaviruses from *N*. *capensis* may identify whether they can be considered as potential recombinant progenitors that may have participated in the emergence of HCoVNL63 [78].

The BtCoVNeo5038/KZN/RSA/2015 betacoronavirus (referred to as BtCoVNeo5038), was detected from the intestine (and the remaining faecal/rectal homogenate) of a *N*. *capensis* collected in the KwaZulu-Natal province. This virus shared 97.7% nucleotide identity to NeoCoV/PML-PHE1 within the compared RdRp gene region. This second variant was detected four years later, confirming the circulation of MERS-related viruses within the host genus. The complete coding genome of BtCoVNeo5038 (30009bp) was sequenced by further amplicon sequencing from 11 amplicons with 1000x coverage on Illumina’s MiSeq platform. The 11 coding regions were in the characteristic coronavirus order, and compared to other lineage C betacoronaviruses (S8 Table). The overall genome similarities between BtCoVNeo5038 and its closest relative, NeoCoV/PML-PHE1, were 97.2% nucleotide identity and 98.5% amino acid similarity. The shared nucleotide identities of all genes were between 96–98.3% (98–99.5% amino acid similarities); with the gene sharing the lowest nucleotide similarity being the spike protein gene (96% nucleotide and 98.7% amino acid similarity). Due to similarities within the seven conserved ORFs required for species demarcation (97.7–100% amino acid similarity) [76], BtCoVNeo5038 may also be assigned to the species, *Middle East respiratory syndrome-related coronavirus*. The species includes the human and camel MERS-CoV strains, as well as the bat viruses NeoCoV/PML-PHE1 and PREDICT/PDF-2108 [4,5]. The latter virus was identified from *Pipistrellus cf*. *hesperidus* collected in Uganda.

BtCoVNeo5038 shares overall nucleotide identities of 85.4–85.5% and concatenated genome amino acid similarities of 88.1–88.2% to selected human and camel strains of MERS-CoV; comparable to similarities of these strains to NeoCoV/PML-PHE1 (S8 Table). Overall genome similarities to MERS-related *Pipistrellus* CoV PREDICT/PDF-2108 [5] were 86.2% nucleotide identity and 91% amino acid similarity. Overall nucleic and amino acid similarities show that the two *Neoromicia* MERS-related coronaviruses share higher genetic similarity to human and camel strains of MERS-CoV (S9 Table).

**Fig 9:**
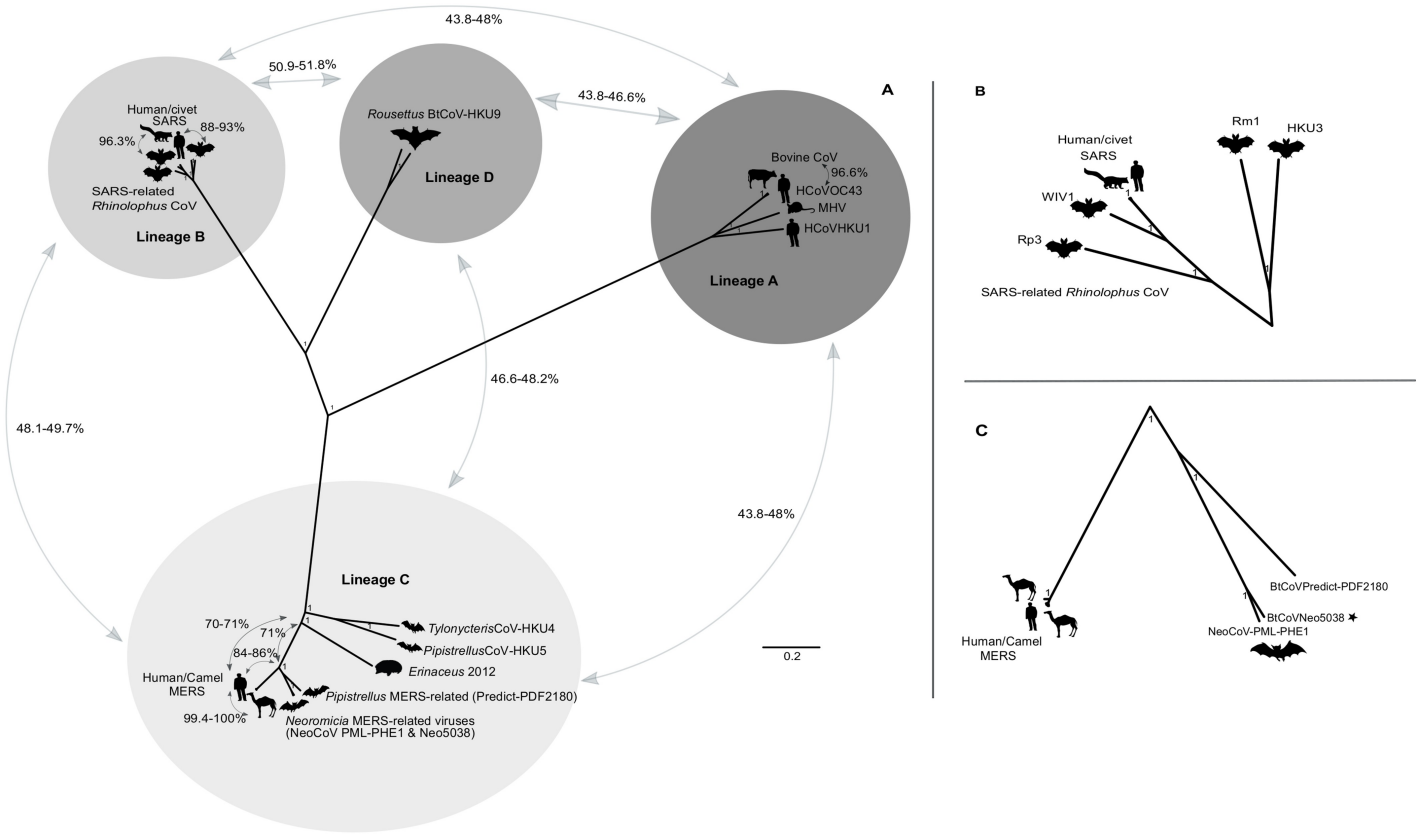
*Betacoronavirus* full genome phylogeny. **A)** The full genome phylogeny of 4 lineages (A-D) of the genus *Betacoronavirus* constructed using BEAST software with the GTR substitution model using invariant sites and gamma distribution. The MCMC chain was set to 15,000,000 generations sampled every 1500 steps, with a 10% burn-in of the first generated trees and displayed as a radial tree in Figtree. The lineages are indicated with clipart images of host species. Also displayed are the averaged pairwise similarities between lineages as well as highlighted similarities between human coronaviruses and related viruses identified in bats (and other animals). **B)** Close-up of the external nodes of the lineage B phylogeny to show relative distances of human and civet SARS-CoV strains and SARS-related *Rhinolophus* strains (WIV1, Rp3, Rm1 and HKU3). **C)** Close-up of the lineage C external nodes depicting the human and camel MERS strains with the bat MERS-related viruses (BtCoVNeo5038 from this study is indicated with a star). Sequence abbreviations and GenBank accession numbers are listed in S10 Table.

All four betacoronavirus lineages (A-D) were included in a full genome phylogenetic comparison (Fig 9A). Similarities of lineage C betacoronaviruses to other lineages range from 43–49% nucleotide identities, and within lineage C similarities are greater than 66%. Comparison of the SARS-CoV lineage (lineage B) to the MERS-CoV lineage, shows shorter phylogenetic distances between the human/civet SARS-CoV and bat associated SARS-related viruses, than between human/camel MERS-CoV and the presently detected bat associated MERS-related viruses (Fig 9B & 9C). At a genetic level, the SARS-related CoV strains circulating in *Rhinolophus* bat hosts share 87.8–95.5% nucleotide identities to the human/civet SARS-CoV strains. Comparatively, the bat MERS-related viruses from the *Neoromicia* and *Pipistrellus* genera share only 83–85.5% nucleotide identities to human/camel MERS-CoV strains.

Dromedary camels have been implicated as the reservoir hosts responsible for MERS-CoV transmission to humans in the Middle East [50]. However, it has also been suggested that the original progenitor viruses of MERS-CoV may have originated as a MERS-related coronavirus harboured by a vespertilionid bat [4,5,80]. The currently identified diversity of bat-borne MERS-related viruses, however, are not sufficiently similar (particularly within the spike protein) to human/camel strains of MERS-CoV to be considered as the possible progenitors. This would mirror the identification of similar SARS-related coronaviruses from *Rhinolophus* hosts. After 11 years of continued surveillance within *Rhinolophus* species, the WIV1 strain capable of utilizing the same binding receptor as human SARS-CoV was finally detected [7]. The finding would lead to the suggestion that through recombination of multiple SARS-related strains, certain SARS-related bat coronaviruses may have been capable of direct human infection [7].

**Fig 10:**
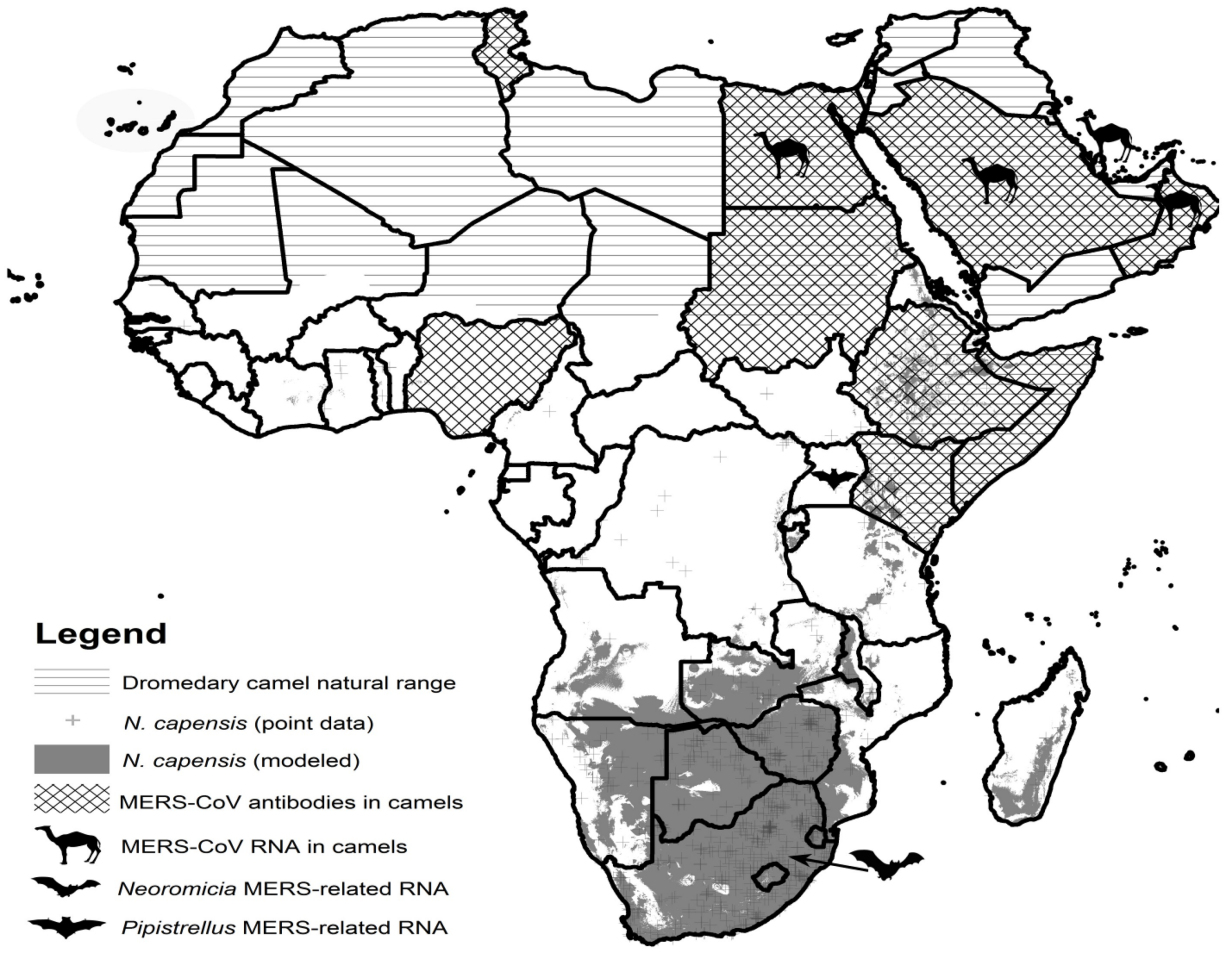
Map of Africa depicting the overlapping distributions of dromedary camels and cape serotine bats as hosts of MERS and related coronaviruses. The map was constructed in ArcMap v.10.4.1. The geographic distribution of dromedary camels are depicted with horizontal lines, with seroprevalence data of MERS antibodies detected from surveillance activities in camels shown with crossed lines. The distributions of *N*. *capensis* were taken from museum collections (point data) and thus extrapolated as modelled data. Clipart images of camels or bats show where viral RNA of MERS and MERS-related strains and have been reported.

Since there is no evidence supporting an equivalent assumption for MERS-related coronaviruses, coronavirus surveillance to identify additional strains or recombinant MERS-related viruses is crucial. Investigations focused in regions where the distributions of the known reservoir, dromedary camels, overlap with known vespertilionid bat hosts (such as *N*. *capensis*), would aid in ascertaining what viruses are circulating. Investigation of camels in these regions would also identify coronaviruses with the potential of recombining with other coronavirus species native to these hosts [81]. Overlapping distributions between dromedary camels and *N*. *capensis*, particularly where MERS antibody prevalence has been reported from camel populations in Africa and the Arabian Peninsula [50–55], indicate the best sampling localities as Sudan, Ethiopia, Somalia and Kenya (Fig 10). Coronavirus surveillance in these regions can be readily conducted with non-invasive sampling of bats and camels (collection of faecal material, oral swabs, and nasal secretions). Improved surveillance may yield additional stains of MERS-related viruses that could be used for isolation attempts in cell lines derived from bats, camels and human tissue. This may in turn allow for functional studies involving isolates that determine host susceptibility and permissibility.

## Conclusions

Multi-pathogen surveillance approaches are integral to pathogen discovery programs that aim to identify potential public health risks, and also increase our knowledge of virus diversity and evolution [82]. The sequence-independent manner utilized by metagenomic high throughput sequencing methodologies enables detection of both known and unknown viral species that may not have been detected with conventional nucleic acid methods. Marked limitations of the metagenomic approach implemented here were highlighted by inadequate sequencing of several viral families, which could subsequently be detected with conventional PCR assays; such as the adenoviruses, herpesviruses and coronaviruses. In spite of the lack of coronavirus contigs produced from the metagenomic data, the complete coding genome of a MERS-related betacoronavirus was still recovered with additional amplicon sequencing. Since the experimental portion of the study was conducted, alternative methods have been suggested that may reduce these biases and improve sequencing results from nonclinical samples with low viral nucleic acid concentrations–such as utilization of the ScriptSeq library kit (Illumina) directly after extraction of RNA [66].

Despite limitations with the metagenomic sequencing output, the study identified a novel *Cyclovirus* species, confirmed MERS-related virus circulation within this host genus, detected diverse adenoviruses and herpesviruses which are widespread among *Neoromicia* populations in South Africa, and determined that adenoviruses seemingly persist within these populations throughout several years. Follow up longitudinal studies can be implemented to confirm this finding and establish the total duration of the viral persistence. The *Neoromicia* adenovirus sequences shared high similarity to those identified in European bats, whereas the *Neoromicia* herpesviruses were much more diverse than previously identified bat-associated viruses. This observation may reflect differences in sampling efforts applied to each viral family. Further investigation of the identified viral families are required to sequence complete genes involved in receptor recognition and attachment to host cells. In the absence of viral isolates, this would allow functional assessment of the receptors utilized for cell entry, enable estimations of their potential to spread to new species, and assess the risks they pose to public or veterinary health. Lastly, the novel sequence data generated from the *Neoromicia* virome can be utilized in assay development for additional nucleic acid detection surveillance activities, to determine the prevalence rates of selected novel viruses.

At present, metagenomic high throughput sequencing may be unsuited for routine viral surveillance practices, as it may be restrictive in terms of sensitivity, incapable of detecting of the complete viral diversity, slow in turn-over time due to extensive bioinformatics data analysis or limited as a result of the high cost of large sequencing volumes. Future improvements to sample preparation and data analysis techniques would be invaluable, and enable these methodologies to be used routinely in strategies for pathogen discovery programs, with the ultimate goal of being aware of high-risk viral species that may be present in wildlife populations.

## Supporting information

S1 File Virome sequence data information from the *Parvoviridae* and *Papillomaviridae* families (with references and figures). (DOCX)

S1 Table *Neoromicia* samples collected to investigate the South African *Neoromicia* virome. (PDF)

S2 Table CLC Genomics workbench bioinformatics workflow parameters. (PDF)

S3 Table *Neoromicia* samples pooled for molecular detection of selected viruses. (PDF)

S4 Table *Alpha*- and *Betacoronavirus* genera hemi-nested RT-PCR primers. (PDF)

S5 Table Pairwise similarities inferred from distance estimations of full length genomes of the *Circoviridae* family. Sequence similarities of viruses in the *Circoviridae* family inferred from estimated evolutionary divergence calculated from pairwise distances. Full genomes were aligned and trimmed to 1075 overlapping positions. All ambiguous positions were removed for each sequence pair. Analyses were conducted in MEGA7 [41].
(PDF)

S6 Table Pairwise similarities inferred from distance estimations of an L gene region between selected *Bunyavirales*. The table shows pairwise sequence similarities inferred from evolutionary divergence estimates of 249 positions of compared *Bunyavirales*. The number of base differences per site from between sequences were converted to percentage of similarities. Standard errors for distance estimates are shown above the diagonal. Codon positions included were 1–3 as well as noncoding. Ambiguous positions were removed for each sequence pair as per pairwise deletion. Estimates were analysed in MEGA7 [41].
(PDF)

S7 Table Pairwise similarities inferred from distance estimations of a 605bp conserved segment of the coronavirus RNA dependent RNA polymerase gene. The table shows pairwise sequence similarities inferred from evolutionary divergence estimates of 605 positions of compared coronaviruses. The number of base differences per site from between sequences were converted to percentage of similarities. The sequences from this study are highlighted in grey and closest similarities to sequences from other studies are indicated in bold. Standard errors for distance estimates are shown above the diagonal in grey text. Codon positions included were 1–3 as well as noncoding and ambiguous positions were removed for each sequence pair as per pairwise deletion. Estimates were analysed in MEGA 7 [41].
(PDF)

S8 Table Genome annotation of BtCoVNeo5038 with similarities to compared lineage C betacoronaviruses. The percentage similarities were inferred from pairwise distance estimates of the base pair and amino acid differences per site for each gene and of the overall genome. The estimates were calculated in MEGA7 [41] using pairwise deletion to treat gaps. All percentage similarities are given in comparison to BtCoVNeoV5038. Accession number of compared betacoronaviruses are listed in order: KC869678.4, KX574227, EF065505.1, EF065509.1, JX869059.2, KF958702.1, KF917527.1, and KJ477102.1.
(PDF)

S9 Table Pairwise similarities inferred from distance estimations between betacoronavirus full genomes. The table shows pairwise similarities inferred from evolutionary divergence estimates of betacoronavirus full genomes. The number of base differences per site between sequences were converted to percentage similarities. Within lineage similarities are indicated in shaded blocks. Standard errors for distance estimates are shown above the diagonal in grey text. Codon positions included were 1–3 as well as noncoding and ambiguous positions were removed for each sequence pair as per pairwise deletion. Estimates were analysed in MEGA 7 [41].
(PDF)

S10 Table Coronavirus strains and Genbank accession numbers of sequences used in the full genome phylogeny. (PDF)
