# Supplementary material for: A metagenomic viral discovery approach identifies potential zoonotic and novel mammalian viruses in Neoromicia bats within South Africa
Source: PLoS One. 2018 Mar 26;13(3):e0194527. doi: 10.1371/journal.pone.0194527 (PMC5868816; doi:10.1371/journal.pone.0194527)
Supplement: S1 File — (DOCX) [file pone.0194527.s001.docx]

**S1 File**: **Virome sequence data from the *Parvoviridae* and *Papillomaviridae***

***Parvoviridae***

Parvoviruses are single stranded DNA viruses with small genomes (4-6kb). There are several genera within the *Parvovirinae* subfamily capable of infecting vertebrates (1). Members of the genus *Dependoparvovirus* require other viruses as ‘helper’ viruses for replication, and thus adenovirus-associated viruses (AAV) are often found co-infecting with adenoviruses. Few studies have identified bat-associated parvoviruses, though of these, many have grouped with dependoparvoviruses. Overall, a large genetic diversity of bat-associated parvoviruses have been documented, with few sharing phylogenetic similarity to viruses circulating in domestic farm animals (2).

A number of contigs assigned as parvoviruses were obtained from the *Neoromicia* virome and the phylogeny constructed with two overlapping contig regions aligning to the capsid gene are shown in supplementary **S1 File Fig. A**. The two overlapping contigs, BtParV/AAV/Neo/1306140 (494bp) and BtParV/AAVNeo/81231 (387bp), shared 80.9% nucleotide identity with each other and 65.2-84% nucleotide identity to members of the genus *Dependoparvovirus*. The similarities among dependoparvovirus sequences are greatest when compared to those from hosts closely related to the genus *Neoromicia*, such and *Myotis* (sharing 81.4-83.8% nucleotide identity) (2). Overall, similarities drop to below 45% when compared to members of the genera *Tetraparovirus*, *Protoparvovirus* and *Parvovirus*. This would be the first report of parvoviruses in African insectivorous bat species.

***
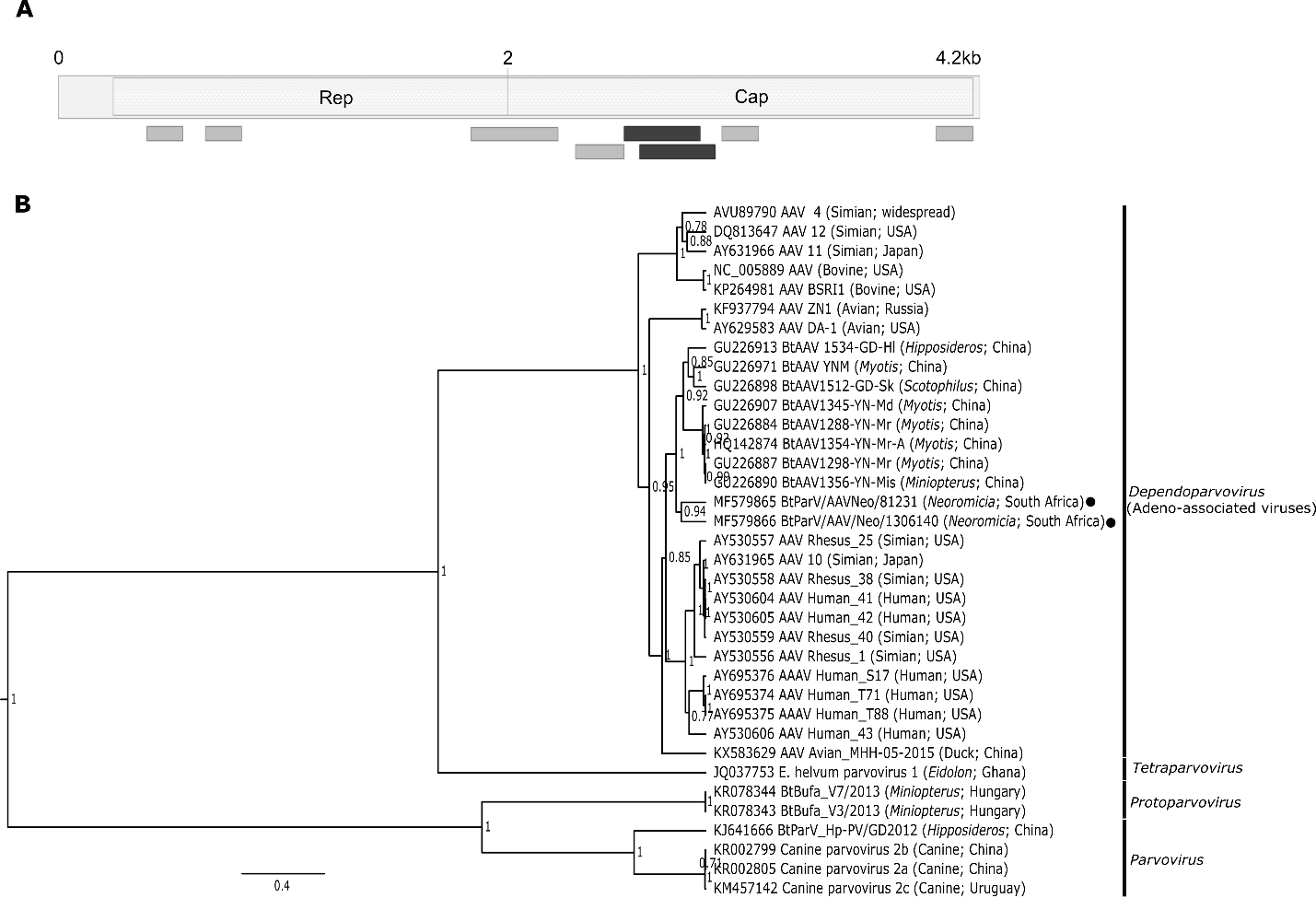
***

**S1 File Figure A**

***Papillomaviridae***

Members of the family *Papillomaviridae* have circular, double stranded, DNA genomes, and often cause benign or malignant neoplastic masses in skin epithelium or mucosal surfaces in a number of vertebrate species (including bats) (3). Papillomaviruses were once considered highly species specific, though recent detections within a number of hosts have shown multiple polyphyletic lineages, suggesting that frequent interspecies transmission events occurred (4). Contigs of the L1 (capsid) and E1 (helicase) region were confirmed with manual inspection of BLASTn results (398bp and 277bp, respectively). The capsid region, which is often targeted in assays as it is generally well conserved, shared greatest pairwise similarities to unclassified bat papillomaviruses from *Eptesicus serotinus* (63-76.2% nucleotide identity), as well as to species in the genus *Kappapapillomavirus* (65.1% nucleotide identity) (S1 File Table A). The E1 region again shared greatest similarity to the another unclassified *Eptesicus serotinus* bat papillomavirus (79% nucleotide identity) (4), followed by between 66.2-66.5% nucleotide identity to bovine papillomaviruses from the genus *Deltapapillomavirus* and feline papillomaviruses from the genus *Lambdapapillomavirus* (S1 File Table B). Intensified surveillance within both the genus *Neoromicia* and other bat species in Southern Africa may identify a larger diversity of these viruses.

**S1 File Table A: Pairwise similarities of a segment of the Papillomaviridae capsid gene inferred from distance estimations**

**S1 File Table B: Pairwise similarities of a segment of the Papillomaviridae E1 gene inferred from distance estimations**

**References of supplementary results**

1. Cotmore SF, Agbandje-McKenna M, Chiorini J a, Mukha D V, Pintel DJ, Qiu J, et al. The family Parvoviridae. Arch Virol. 2014;159(5):1239–47.

2. Li Y, Ge X, Hon C-C, Zhang H, Zhou P, Zhang Y, et al. Prevalence and genetic diversity of adeno-associated viruses in bats from China. J Gen Virol. 2010 Oct;91(Pt 10):2601–9.

3. Rector A, Mostmans S, Doorslaer K Van, Mcknight CA, Maes RK, Wise AG, et al. Genetic characterization of the first chiropteran papillomavirus, isolated from a basosquamous carcinoma in an Egyptian fruit bat: The Rousettus aegyptiacus papillomavirus type 1. Vet J. 2006;117:267–75.

4. García-Pérez R, Ibáñez C, Godínez JM, Aréchiga N, Garin I, Pérez-Suárez G, et al. Novel papillomaviruses in free-ranging Iberian bats: no virus-host co-evolution, no strict host specificity, and hints for recombination. Genome Biol Evol. 2014;6(1):94–104.
