# Supplementary material for: A metagenomic viral discovery approach identifies potential zoonotic and novel mammalian viruses in Neoromicia bats within South Africa
Source: PLoS One. 2018 Mar 26;13(3):e0194527. doi: 10.1371/journal.pone.0194527 (PMC5868816; doi:10.1371/journal.pone.0194527)
Supplement: S1 Table — (PDF) [file pone.0194527.s002.pdf]

S1 Table: *Neoromicia* samples collected and pooled to investigate the South African *Neoromicia* virome

Laboratory number	Museum number	Field Number	Species confirmation	Gender	Collection date	Site	Province	Coordinates		Available specimens		
								Y	X	Faecal	Rectum	Intestines
UP 0161	TM48032	ECJS19/2007	*Neoromicia capensis*	M	12/14/2007	Taung World Heritage Site	North West	-27.61543	24.63005	-	**+**	-
UP 0165	TM48042	ECJS36/2007	*Neoromicia capensis*	F	12/15/2007	Taung World Heritage Site	North West	-27.61543	24.63005	-	**+**	-
UP 0166	TM48046	ECJS45/2007	*Neoromicia capensis*	F	12/15/2007	Taung World Heritage Site	North West	-27.61543	24.63005	-	**+**	-
UP 0167	TM48043	ECJS38/2007	*Neoromicia capensis*	F	12/15/2007	Taung World Heritage Site	North West	-27.61543	24.63005	-	**+**	-
UP 0168	TM48044	ECJS39/2007	*Neoromicia capensis*	F	12/15/2007	Taung World Heritage Site	North West	-27.61543	24.63005	-	**+**	-
UP 0169	TM48045	ECJS42/2007	*Neoromicia capensis*	M	12/15/2007	Taung World Heritage Site	North West	-27.61543	24.63005	-	**+**	-
UP 0204	TM48028	ECJS15/2007	*Neoromicia capensis*	M	2/12/2007	Madikwe Game Reserve/ Kalkfontein Farm 111KP	North West	-24.79699	26.30092	-	**+**	-
UP 0206	TM48030	ECJS17/2007	*Neoromicia capensis*	M	2/12/2007	Madikwe Game Reserve/ Kalkfontein Farm 111KP	North West	-24.79699	26.30092	**+**	+	-
UP 0207	TM48031	ECJS18/2007	*Neoromicia capensis*	M	2/12/2007	Madikwe Game Reserve/ Kalkfontein Farm 111KP	North West	-24.79699	26.30092	-	**+**	-
UP 1012	TM48660	18	*Neoromicia capensis*	F	10/12/2010	Kgaswane Nature Reserve/ Baviaanskrans Farm 308JQ	North West	-25.73903	27.23011	**+**	+	+
UP 1021	TM48661	15	*Neoromicia capensis*	M	10/12/2010	Kgaswane Nature Reserve/ Baviaanskrans Farm 308JQ	North West	-25.73903	27.23011	**+**	+	+
UP 0724	TM48505	ECJS-12/2010	*Neoromicia nana*	F	2/1/2010	Pafuri, Kruger National Park	Limpopo	-22.42151	31.2238	-	-	**+**
UP 0725	TM48506	ECJS-13/2010	*Neoromicia zuluensis*	F	2/1/2010	Pafuri, Kruger National Park	Limpopo	-22.42151	31.2238		**+**	+
UP 0727	TM48508	ECJS-17/2010	*Neoromicia nana*	F	2/1/2010	Pafuri, Kruger National Park	Limpopo	-22.42151	31.2238	**+**	+	+
UP 0728	TM48509	ECJS-18/2010	*Neoromicia nana*	F	2/1/2010	Pafuri, Kruger National Park	Limpopo	-22.42151	31.2238	**+**	+	+
UP 0760	TM48533	-	*Neoromicia nana*	M	2/3/2010	Pafuri, Kruger National Park	Limpopo	-22.34657	31.11595	**+**	+	-
UP 0761	TM48534	-	*Neoromicia nana*	F	2/3/2010	Pafuri, Kruger National Park	Limpopo	-22.34657	31.11595	-	**+**	-
UP 0762	TM48535	-	*Neoromicia cf. helios*	M	2/3/2010	Pafuri, Kruger National Park	Limpopo	-22.34657	31.11595	**+**	-	+
UP 0763	TM48536	-	*Neoromicia cf. helios*	F	2/3/2010	Pafuri, Kruger National Park	Limpopo	-22.34657	31.11595	**+**	-	-
UP 0764	TM48537	-	*Neoromicia cf. helios*	F	2/3/2010	Pafuri, Kruger National Park	Limpopo	-22.34657	31.11595	**+**	+	+
UP 0915	TM48570	10	*Neoromicia capensis*	F	11/8/2010	Pafuri, Kruger National Park	Limpopo	-22.42151	31.2238	**+**	-	+
UP 0918	-	6	*Neoromicia nana*	F	11/8/2010	Pafuri, Kruger National Park	Limpopo	-22.42151	31.2238	**+**	-	-
UP 0919	-	7	*Neoromicia nana*	F	11/8/2010	Pafuri, Kruger National Park	Limpopo	-22.42151	31.2238	**+**	-	-
UP 0920	TM48571	1	*Neoromicia nana*	M	11/9/2010	Pafuri, Kruger National Park	Limpopo	-22.42151	31.2238	-	**+**	+
UP 0921	-	3	*Neoromicia cf. helios*	F	11/9/2010	Pafuri, Kruger National Park	Limpopo	-22.42151	31.2238	-	**+**	+
UP 0922	TM48572	4	*Neoromicia nana*	M	11/9/2010	Pafuri, Kruger National Park	Limpopo	-22.42151	31.2238	-	**+**	+
UP 0923	TM48573	6	*Neoromicia nana*	M	11/9/2010	Pafuri, Kruger National Park	Limpopo	-22.42151	31.2238	-	**+**	-
UP 0931	TM48578	2B	*Neoromicia cf. helios*	F	11/10/2010	Pafuri, Kruger National Park	Limpopo	-22.42601	31.29873	-	**+**	+
UP 0952	TM48587	7B	*Neoromicia cf. helios*	F	11/12/2010	Pafuri, Kruger National Park	Limpopo	-22.34657	31.11595	**+**	+	+
UP 0961	TM48596	25A	*Neoromicia zuluensis*	F	11/12/2010	Pafuri, Kruger National Park	Limpopo	-22.34657	31.11595	**+**	+	+
UP 0962	TM48597	22A	*Neoromicia nana*	F	11/12/2010	Pafuri, Kruger National Park	Limpopo	-22.34657	31.11595	**+**	+	+
UP 0963	TM48598	18B	*Neoromicia capensis*	F	11/12/2010	Pafuri, Kruger National Park	Limpopo	-22.34657	31.11595	**+**	+	+
UP 1787	TM49186	37	*Neoromicia capensis*	M	1/19/2013	Randstephne Farm455KQ	Limpopo	-24.59751	27.67145	**+**	+	+
UP 1798	-	1	*Neoromicia zuluensis*	M	1/21/2013	Donkerpoort Farm448KQ	Limpopo	-24.59306	27.67956	**+**	-	-
UP 1799	-	2	*Neoromicia capensis*	M	1/21/2013	Donkerpoort Farm448KQ	Limpopo	-24.59306	27.67956	**+**	-	-
UP 1800	-	3	*Neoromicia capensis*	M	1/21/2013	Donkerpoort Farm448KQ	Limpopo	-24.59306	27.67956	**+**	-	-
UP 3887	TM49146	-	*Neoromicia nana*	M	5/2/2014	Mahune site, Gamafefe	Limpopo	-24.61799	27.65235	**+**	+	+
UP 3916	TM49147	-	*Neoromicia zuluensis*	F	5/2/2014	Mahune site, Gamafefe	Limpopo	-24.61799	27.65235	**+**	+	+
UP 4965	TM49188	-	*Neoromicia capensis*	M	2/26/2015	Randstephne Farm 455 KQ	Limpopo	-24.59751	27.67145	**+**	-	-
UP 4972	TM49189	-	*Neoromicia capensis*	F	2/28/2015	Jonker Trust Farm/ Zandspruit 449KQ	Limpopo	-24.61737	27.68773	**+**	-	+
UP 5013	TM49069	-	*Neoromicia capensis*	M	3/3/2015	Jonker Trust Farm/ Zandspruit 449KQ	Limpopo	-24.625197	27.69787	**+**	+	+
UP 5014	TM49190	-	*Neoromicia capensis*	F	3/3/2015	Jonker Trust Farm/ Zandspruit 449KQ	Limpopo	-24.625197	27.69787	**+**	+	+
UP 1369	-	-	*Neoromicia capensis*	M	9/24/2011	Free Me rehab	Gauteng	-26.030063	28.040890	-	+	-
UP 3921	TM49169	ECJS-09/2014	*Neoromicia capensis*	F	1/1/2014	Telperion NR/ Sterkfontein Farm 495JR	Mpumalanga	-25.7113	29.01755	-	**+**	+
UP 3922	TM49170	ECJS-10/2014	*Neoromicia capensis*	M	1/1/2014	Telperion NR/ Sterkfontein Farm 495JR	Mpumalanga	-25.72596	28.99048	-	**+**	+
UP 3923	TM49171	ECJS-11/2014	*Neoromicia capensis*	M	1/1/2014	Telperion NR/ Sterkfontein Farm 495JR	Mpumalanga	-25.72596	28.99048	-	**+**	+
UP 4813	TM49191	2	*Neoromicia capensis*	F	1/24/2015	Leeudraai Farm221 JR	Gauteng	-25.37081	28.56614	**+**	+	+
UP 4814	TM49192	6	*Neoromicia capensis*	F	1/24/2015	Leeudraai Farm221 JR	Gauteng	-25.37081	28.56614	-	**+**	+
UP 4815	-	8	*Neoromicia capensis*	F	1/24/2015	Leeudraai Farm221 JR	Gauteng	-25.37081	28.56614	**+**	-	-
UP 4816	TM49193	10	*Neoromicia capensis*	M	1/24/2015	Leeudraai Farm221 JR	Gauteng	-25.37081	28.56614	**+**	+	+
UP 4817	-	1	*Neoromicia capensis*	M	1/24/2015	Leeudraai Farm221 JR	Gauteng	-25.37081	28.56614	**+**	-	-
UP 4819	-	4	*Neoromicia capensis*	F	1/24/2015	Leeudraai Farm221 JR	Gauteng	-25.37081	28.56614	**+**	-	-
UP 4820	-	5	*Neoromicia capensis*	M	1/24/2015	Leeudraai Farm221 JR	Gauteng	-25.37081	28.56614	**+**	-	-
UP 4823	-	9	*Neoromicia capensis*	F	1/24/2015	Leeudraai Farm221 JR	Gauteng	-25.37081	28.56614	**+**	-	-
UP 4825	-	-	*Neoromicia capensis*	F	1/24/2015	Leeudraai Farm221 JR	Gauteng	-25.37081	28.56614	**+**	-	-
UP 5036	TM49182	ECJS-114/2015	*Neoromicia capensis*	M	4/20/2015	Wakefield Farm2369/ ptn 13	KwaZulu-Natal	-29.48694	29.89806	**+**	+	+
UP 5038	TM49183	ECJS-116/2015	*Neoromicia capensis*	M	4/20/2015	Wakefield Farm2369/ ptn 13	KwaZulu-Natal	-29.48694	29.89806	**+**	+	+
UP 5304	TM49194	-	*Neoromicia capensis*	F	2/5/2015	Welgevonden Farm 969	KwaZulu-Natal	-29.331947	30.27753	-	**+**	+

Specimen material in bold indicate the sample type used for the Illumina sequencing
