# Supplementary material for: A metagenomic viral discovery approach identifies potential zoonotic and novel mammalian viruses in Neoromicia bats within South Africa
Source: PLoS One. 2018 Mar 26;13(3):e0194527. doi: 10.1371/journal.pone.0194527 (PMC5868816; doi:10.1371/journal.pone.0194527)
Supplement: S2 Table — (PDF) [file pone.0194527.s003.pdf]

S2 Table: CLC Genomics workbench bioinformatics workflow parameters

Step 1: Initial trimming parameters						
**Quality trimming**						
Ambiguous trim	yes					
Ambiguous limit	2					
Quality limit	0.05					
Quality Trim	yes					
**Sequence to Trim**	**Strand**	**Action**	**Mismatch**	**Gapcost**	**Cutoff**	**Sequence**
TruSeq-adapter-1	Both	Remove adapter	2	3	10	GGGGTAGTGTGGATCCTCCTCTAGGCAGTTGGGTTATTCTAGAAGCAGATGTGTTGGCTGTTTCTGAAACTCTGGAAAA
TruSeq-adapter-3	Both	Remove adapter	2	3	10	CAACAGCCGGTCAAAACATCTGGAGGGTAAGCCATAAACACCTCAACAGAAAA
PCR-primer-1	Both	Remove adapter	2	3	10	CGATAACTTCGTATAATGTATGCTATACGAAGTTATTACG
PCR-primer-2	Both	Remove adapter	2	3	10	GCATAACTTCGTATAGCATACATTATACGAAGTTATACGA
Nextera-primer-adapter-1	Both	Remove adapter	2	3	10	GATCGGAAGAGCACACGTCTGAACTCCAGTCAC
Nextera-primer-adapter-2	Both	Remove adapter	2	3	10	GATCGGAAGAGCGTCGTGTAGGGAAAGAGTGT
Nextera-junction-adapter-1	Both	Remove adapter	2	3	10	CTGTCTCTTATACACATCTAGATGTGTATAAGAGACAG
SmartSeq-adapter-1	Both	Remove adapter	2	3	10	AATGATACGGCGACCACCGAGATCTACACTTCGCTACAGGACGCTGTTCCGTTCTATGGG
SmartSeq-adapter-2	Both	Remove adapter	2	3	10	ACACTTCGCTACAGGACGCTGTTCCGTTCTATGGG
SmartSeq-adapter-3	Both	Remove adapter	2	3	10	CAGACGTGTGCTCTTCCGATCT
5'PolyG	Plus	Remove adapter	2	3	-	G^{150}
3'PolyG	Minus	Remove adapter	2	3	-	C^{150}
PCR-20mer	Plus	Remove adapter	2	3	10	GACCATCTAGCGACCTCCCA
**Filtering on length**						
Max nr of nt in reads	1000					
Min nr of nt in reads	20					
Short reads discarded	yes					
Step 2: Map to host parameters						
**Input host genomes/scaffolds downloaded from NCBI: *Myotis brandtii* assembly no. GCF_00412655.1**						
Masking mode	no					
Mismatch cost	2					
Insertion cost	3					
Deletion cost	3					
Length fraction	0.7					
Similarity fraction	0.9					
Global alignment	no					
Color error cost	3					
Auto detect paired distances	yes					
Non-specific match handling	Map randomly					
**Outputs:**						
Report	produced					
Read mapping	Host NGS reads					
Unmapped reads	Non-host NGS reads					
**Remove PhiX sequencing control**						
Same parameters as mapping to host						
**Step 3: CLC de novo assembly**						
**Map reads to contigs**						
Mapping mode	Map reads back to contigs (slow)					
Update contigs	yes					
**Mapping options**						
Mismatch cost	2					
Insertion cost	3					
Deletion cost	3					
Length fraction	0.5					
Similarity fraction	0.8					
Alignment mode	local					
Color space error cost	3					
Create list of unmapped reads	yes					
**Graph parameters**						
Automatic bubble size	yes					
Bubble size	50					
Automatic word size	yes					
Word size	20					
**Contig length**						
minimun contig length	200					
**Paired reads**						
Perform scaffolding	yes					
Autodetect paired distances	yes					
**Outputs:**						
Report	yes					
Assembly with Mapping	NGS contig output					
Unmapped reads	Left over reads not included in the assembly					
