# Supplementary material for: A metagenomic viral discovery approach identifies potential zoonotic and novel mammalian viruses in Neoromicia bats within South Africa
Source: PLoS One. 2018 Mar 26;13(3):e0194527. doi: 10.1371/journal.pone.0194527 (PMC5868816; doi:10.1371/journal.pone.0194527)
Supplement: S3 Table — (PDF) [file pone.0194527.s004.pdf]

**S3 Table: *Neoromicia* virome samples available for molecular detection**

| Specimen | | | Confirmation of species identification | Province | Feacal | Rectum | Intestines |
|---|---|---|---|---|---|---|---|
| **North West** | | | | | | | |
| 1 | Pool | UP 0204 | *Neoromicia capensis* | North West | - | **+** | - |
| | | UP 0206 | *Neoromicia capensis* | North West | - | + | - |
| | | UP 0207 | *Neoromicia capensis* | North West | - | **+** | - |
| 2 | Pool | UP 1012 | *Neoromicia capensis* | North West | - | - | + |
| | | UP 1021 | *Neoromicia capensis* | North West | - | - | + |
| **Gauteng** | | | | | | | |
| 3 | Not Pooled | UP 1369 | *Neoromicia capensis* | Gauteng | - | **+** | + |
| 5 | Pool | UP 4813 | *Neoromicia capensis* | Gauteng | + | - | + |
| | | UP 4814 | *Neoromicia capensis* | Gauteng | - | **+** | - |
| | | UP 4816 | *Neoromicia capensis* | Gauteng | + | - | + |
| **Mpumalanga** | | | | | | | |
| 4 | Pool | UP 3921 | *Neoromicia capensis* | Mpumalanga | - | **+** | + |
| | | UP 3922 | *Neoromicia capensis* | Mpumalanga | - | **+** | + |
| | | UP 3923 | *Neoromicia capensis* | Mpumalanga | - | **+** | + |
| **KwaZulu-Natal** | | | | | | | |
| 6 | Pool | UP 5036 | *Neoromicia capensis* | KwaZulu-Natal | **+** | - | + |
| | | UP 5038 | *Neoromicia capensis* | KwaZulu-Natal | **+** | - | + |
| | | UP 5304 | *Neoromicia capensis* | KwaZulu-Natal | - | **+** | + |
| **Limpopo** | | | | | | | |
| 7 | Not Pooled | UP 0725 | *Neoromicia zuluensis* | Limpopo | - | - | + |
| 8 | Pool | UP 0724 | *Neoromicia nana* | Limpopo | - | - | **+** |
| | | UP 0727 | *Neoromicia nana* | Limpopo | **+** | - | + |
| | | UP 0728 | *Neoromicia nana* | Limpopo | **+** | - | + |
| 9 | Pool | UP 0760 | *Neoromicia nana* | Limpopo | + | + | - |
| | | UP 0761 | *Neoromicia nana* | Limpopo | - | **+** | - |
| 10 | Pool | UP 0762 | *Neoromicia helios* | Limpopo | **+** | - | + |
| | | UP 0763 | *Neoromicia helios* | Limpopo | **+** | - | - |
| | | UP 0764 | *Neoromicia helios* | Limpopo | **+** | - | + |
| 11 | Not Pooled | UP 0915 | *Neoromicia capensis* | Limpopo | + | - | + |
| 12 | Pool | UP 0920 | *Neoromicia nana* | Limpopo | - | | + |
| | | UP 0922 | *Neoromicia nana* | Limpopo | - | **+** | + |
| | | UP 0923 | *Neoromicia nana* | Limpopo | - | **+** | - |
| 13 | Pool | UP 0921 | *Neoromicia helios* | Limpopo | - | - | + |
| | | UP 0931 | *Neoromicia helios* | Limpopo | - | **+** | + |
| | | UP 0952 | *Neoromicia helios* | Limpopo | - | - | + |
| 14 | Pool | UP 0961 | *Neoromicia zuluensis* | Limpopo | **+** | - | + |
| | | UP 0963 | *Neoromicia capensis* | Limpopo | **+** | - | + |
| | | UP 0962 | *Neoromicia nana* | Limpopo | - | - | + |
| 16 | Not Pooled | UP 1787 | *Neoromicia capensis* | Limpopo | - | - | + |
| 17 | Pool | UP 3887 | *Neoromicia nana* | Limpopo | - | - | + |
| | | UP 3916 | *Neoromicia zuluensis* | Limpopo | **+** | - | + |
| 18 | Pool | UP 4965 | *Neoromicia capensis* | Limpopo | **+** | - | - |
| | | UP 4972 | *Neoromicia capensis* | Limpopo | - | - | + |
| 19 | Pool | UP 5013 | *Neoromicia capensis* | Limpopo | **+** | - | + |
| | | UP 5014 | *Neoromicia capensis* | Limpopo | **+** | - | + |

*Specimen material in bold indicates availability of remaining homogenates previously utilized for Illumina sequencing.*
