# Supplementary material for: A metagenomic viral discovery approach identifies potential zoonotic and novel mammalian viruses in Neoromicia bats within South Africa
Source: PLoS One. 2018 Mar 26;13(3):e0194527. doi: 10.1371/journal.pone.0194527 (PMC5868816; doi:10.1371/journal.pone.0194527)
Supplement: S4 Table — (PDF) [file pone.0194527.s005.pdf]

S4 Table: *Alpha-* and *Betacoronavirus* heminested RT-PCR primers

First round primers	5'-3' Sequence	Amplicon size
**P1Alpha/Beta_For2016**	ATGGGHTGGGAYTAYCCHAARTGYGA	442-443bp
**P1Alpha_Rev2016**	CCRTCATCWGAIARDATCATCAT	
**P1Beta_Rev2016**	CATCRTCASDIARDATCATCAT	
**Nested PCR primers**		
**P2ALPHA_For2016**	TYTAYNTNAARCCWGGTGG	266-268bp
**P2BETA_For2016**	TAYGTIAARCCWGGHGGIAC	
