# Supplementary material for: A metagenomic viral discovery approach identifies potential zoonotic and novel mammalian viruses in Neoromicia bats within South Africa
Source: PLoS One. 2018 Mar 26;13(3):e0194527. doi: 10.1371/journal.pone.0194527 (PMC5868816; doi:10.1371/journal.pone.0194527)
Supplement: S5 Table — Sequence similarities of viruses in the Circoviridae family inferred from estimated evolutionary divergence calculated from pairwise distances. Full genomes were aligned and trimmed to 1075 overlapping positions. All ambiguous positions were removed for each sequence pair. Analyses were conducted in MEGA7 [41]. (PDF) [file pone.0194527.s006.pdf]

S5 Table: Pairwise similarities inferred from distance estimations of full length genomes of the *Circoviridae* family

Accession	Circoviruses as comparison	1	2	3	4	5	6	7	8	9	10	11	12	13	14	15	16	17	18	19	20	21	22	23	24	25	26	27	28	29	30	31	32	Genera
this study	NeoCycloV-1 (19681/RSA)		0.013	0.013	0.013	0.013	0.013	0.013	0.014	0.013	0.013	0.013	0.012	0.012	0.013	0.013	0.012	0.013	0.013	0.014	0.013	0.012	0.014	0.013	0.013	0.013	0.013	0.012	0.012	0.011	0.013	0.012	0.012	
GQ4048455	CyclovirusPK5034	64.9		0.014	0.013	0.013	0.013	0.013	0.014	0.012	0.013	0.013	0.012	0.013	0.012	0.013	0.013	0.013	0.013	0.013	0.013	0.012	0.013	0.014	0.014	0.013	0.014	0.013	0.013	0.012	0.013	0.012	0.013	
GQ404846	CyclovirusPK5222	60.5	58.3		0.012	0.013	0.014	0.013	0.012	0.012	0.013	0.013	0.013	0.013	0.013	0.014	0.012	0.013	0.013	0.013	0.012	0.012	0.013	0.012	0.012	0.013	0.013	0.013	0.013	0.012	0.014	0.013	0.013	
KJ641712	BtCV_BtRp-CV-3/GD2012	59.8	57.8	57.6		0.013	0.013	0.012	0.013	0.012	0.012	0.013	0.013	0.012	0.013	0.012	0.013	0.013	0.013	0.013	0.013	0.012	0.014	0.013	0.014	0.012	0.013	0.013	0.013	0.013	0.013	0.012	0.013	
HQ738637	BtCV/USA/2009-TB	58.8	57.9	56.6	55.4		0.013	0.014	0.012	0.013	0.014	0.013	0.012	0.012	0.012	0.013	0.012	0.014	0.013	0.014	0.013	0.014	0.013	0.012	0.013	0.013	0.012	0.013	0.013	0.012	0.012	0.012	0.012	
KM382269	BtCVPOA/2012/II	58.7	57.6	58.2	57.9	55.5		0.013	0.014	0.013	0.014	0.013	0.014	0.013	0.013	0.013	0.013	0.013	0.013	0.013	0.012	0.013	0.012	0.014	0.014	0.012	0.013	0.013	0.013	0.013	0.014	0.012	0.012	
KM382270	BtCVPOA/2012/VI	56.2	59.5	54.4	57.9	56.2	56.5		0.013	0.013	0.013	0.013	0.012	0.012	0.013	0.013	0.014	0.012	0.013	0.013	0.012	0.013	0.013	0.013	0.013	0.012	0.011	0.012	0.012	0.012	0.013	0.012	0.012	
NC_014928	CyVPKgoat11/PAK/2009	58.9	55.9	65.7	54.2	56.7	58.0	52.8		0.013	0.013	0.013	0.014	0.013	0.013	0.014	0.013	0.014	0.013	0.014	0.013	0.013	0.013	0.012	0.012	0.012	0.013	0.013	0.012	0.012	0.014	0.013	0.012	
KC512920	DfCyV-8_AU-DFB007B-2010	54.8	56.1	58.2	56.3	53.4	57.2	62.8	55.6		0.013	0.012	0.014	0.013	0.012	0.013	0.013	0.012	0.013	0.014	0.013	0.012	0.013	0.013	0.013	0.012	0.012	0.013	0.014	0.013	0.013	0.013	0.013	
KC512918	DfCyV-6_US-DFKWGX-2012	57.4	55.0	55.7	53.0	53.7	57.1	56.9	55.0	55.0		0.014	0.013	0.012	0.013	0.012	0.014	0.013	0.012	0.013	0.013	0.013	0.012	0.013	0.013	0.012	0.012	0.013	0.012	0.012	0.013	0.013	0.012	
KF031470	HumanCVVN_ps1	44.2	43.8	44.6	44.4	44.6	45.6	44.8	43.7	44.9	46.9		0.013	0.013	0.013	0.013	0.013	0.013	0.013	0.013	0.012	0.013	0.013	0.012	0.012	0.012	0.013	0.012	0.012	0.013	0.013	0.013	0.012	
NC_014930	CyV_NGchicken15/NGA/2009	58.6	56.9	59.5	55.9	56.0	55.2	53.3	58.7	54.3	54.2	43.9		0.012	0.013	0.014	0.013	0.013	0.012	0.013	0.013	0.013	0.013	0.012	0.012	0.013	0.013	0.013	0.013	0.012	0.013	0.012	0.012	*Cyclovirus*
GQ404849	CyVChimp11	58.6	57.5	60.9	56.1	59.1	55.4	54.5	60.3	57.0	54.1	42.7	63.9		0.014	0.013	0.013	0.013	0.012	0.014	0.013	0.013	0.012	0.012	0.012	0.012	0.012	0.013	0.013	0.012	0.013	0.012	0.012	
NC_014927	CyVPKgoat21/PAK/2009	47.5	46.0	47.4	47.0	46.2	46.4	47.0	45.2	45.7	49.5	65.6	45.6	45.8		0.013	0.012	0.014	0.013	0.014	0.012	0.012	0.013	0.012	0.012	0.012	0.012	0.012	0.012	0.012	0.013	0.012	0.012	
JF938080	BtCVZS/China/2011YN-BtCV-3	56.3	55.8	58.3	56.3	52.7	57.8	57.6	57.2	58.3	68.1	45.6	55.8	56.2	48.0		0.013	0.013	0.013	0.014	0.012	0.012	0.013	0.014	0.014	0.012	0.012	0.012	0.012	0.012	0.013	0.013	0.012	
JF938081	BtCVZS/China/2011YN-BtCV-4	56.1	60.2	61.1	57.7	60.0	56.0	57.1	58.6	55.3	54.0	43.7	60.6	62.1	45.8	56.5		0.013	0.013	0.014	0.013	0.013	0.013	0.013	0.013	0.012	0.012	0.012	0.012	0.013	0.013	0.013	0.013	
KJ641717	BtCVBtMspp.-CV/GD2012	54.5	55.1	55.2	54.6	52.9	60.4	54.6	54.1	53.9	53.6	45.1	54.2	52.2	45.7	55.7	53.9		0.014	0.013	0.012	0.013	0.014	0.013	0.013	0.012	0.012	0.014	0.013	0.012	0.012	0.012	0.012	
KJ641734	BtCVBtVS-CV/SC2013	54.4	54.2	53.8	56.3	53.9	57.0	63.7	53.0	59.7	55.9	46.0	54.0	55.0	46.6	56.3	55.8	53.6		0.013	0.012	0.013	0.007	0.013	0.014	0.012	0.013	0.013	0.013	0.012	0.014	0.012	0.012	
KJ641728	BtCVBtPa-CV-2/NX2013	53.3	53.5	55.4	52.6	50.3	54.3	54.7	54.1	55.9	59.5	44.4	51.9	51.8	46.2	58.1	53.3	52.4	55.1		0.013	0.013	0.014	0.013	0.012	0.012	0.012	0.012	0.013	0.011	0.012	0.012	0.012	
KJ641720	BtCVBtTp-CV-2/GX2012	54.0	55.4	55.4	54.6	53.8	56.6	65.2	53.6	61.0	59.0	44.8	53.9	54.0	45.6	57.4	57.9	51.8	67.3	55.8		0.013	0.013	0.013	0.013	0.011	0.013	0.013	0.013	0.012	0.013	0.012	0.013	
JF938079	BtCVZS/China/2011YN-BtCV-2	57.4	57.0	59.0	56.7	58.5	57.3	56.4	57.9	54.6	54.9	43.2	59.7	59.3	47.6	56.3	61.5	54.4	55.8	52.7	58.0		0.013	0.013	0.013	0.012	0.013	0.013	0.013	0.012	0.013	0.013	0.011	
KJ641715	BtCVBtRp-CV-52/GD2012	54.6	54.3	54.2	55.9	53.7	56.9	63.4	54.0	60.0	56.7	45.9	54.3	55.1	46.7	56.2	56.1	54.1	93.7	55.0	67.2	55.3		0.013	0.014	0.012	0.013	0.013	0.013	0.012	0.014	0.012	0.013	
NC_021206	BtCVXOR7	38.1	40.2	37.2	36.1	38.2	38.7	38.0	36.7	37.9	38.8	36.6	37.3	37.7	36.9	37.3	37.2	39.6	38.1	35.4	38.9	38.4	37.4		0.013	0.013	0.014	0.012	0.013	0.013	0.014	0.012	0.013	
NC_001792	PorcineCV1	40.8	40.5	39.0	38.6	40.9	40.7	38.1	39.9	37.4	39.1	37.7	39.6	38.9	36.7	38.3	37.9	39.1	37.8	36.9	39.3	39.0	37.6	66.1		0.012	0.014	0.013	0.012	0.013	0.013	0.012	0.012	
NC_002361	ColumbidCV	39.2	41.3	38.5	37.5	38.0	40.1	38.3	38.7	37.4	39.2	37.0	37.8	39.0	36.2	39.7	39.4	38.7	39.0	39.2	37.5	39.7	39.0	44.1	43.6		0.013	0.013	0.013	0.012	0.012	0.012	0.012	
NC_003410	CanaryCV	38.8	40.6	38.0	38.7	38.9	39.0	38.2	39.1	36.5	38.1	36.3	38.4	36.5	36.3	39.3	38.1	39.2	39.0	38.1	39.2	39.0	38.7	43.9	43.7	59.3		0.012	0.014	0.013	0.013	0.012	0.012	
NC_008522	FinchCV	38.4	38.6	38.6	38.8	39.5	38.8	38.2	39.3	36.2	38.8	35.8	38.3	37.1	35.8	38.4	37.8	38.6	38.8	38.3	39.2	38.6	39.4	45.0	45.4	60.5	66.3		0.013	0.013	0.013	0.013	0.011	*Circovirus*
NC_001944	Beak & feather disease virus	37.7	38.5	38.8	37.7	38.3	39.6	38.3	38.9	36.9	39.2	35.0	37.9	37.1	35.3	38.0	37.0	39.3	41.4	39.5	39.7	40.9	41.3	44.3	42.8	59.8	54.9	56.1		0.013	0.013	0.013	0.013	
JX863737	BtCVXOR	41.6	41.3	39.7	40.9	40.5	41.8	41.2	40.3	39.9	42.7	38.4	41.6	41.2	38.7	41.0	38.9	40.2	41.4	42.0	41.0	41.4	40.9	42.1	44.3	41.7	42.6	42.5	42.3		0.013	0.012	0.013	
JQ814849	BtCVRh.ferrumequinumCV1	39.6	40.0	38.0	37.6	38.7	40.7	40.3	39.2	38.5	40.8	36.9	38.1	37.9	34.8	39.0	39.0	37.9	40.7	39.0	40.2	40.8	40.4	41.3	43.2	41.3	42.6	41.0	43.2	44.3		0.011	0.012	
JF938078	BtCVZS/China/2011YN-BtCV-1	36.4	35.9	35.2	33.4	35.8	36.7	35.5	35.1	35.4	39.1	34.6	35.8	35.3	35.5	35.7	34.8	32.0	34.4	36.3	34.6	34.7	34.8	33.6	32.9	33.3	33.1	32.6	32.3	34.8	32.9		0.012	*Unclassified*
KM382272	BtCVPOA/2012/V	34.7	32.6	32.9	35.3	35.4	35.6	32.6	32.7	34.3	32.8	32.7	32.3	32.3	33.4	34.1	32.3	32.9	32.9	33.1	32.7	35.1	33.4	29.2	29.1	29.9	29.6	29.1	29.2	31.0	31.8	29.4		
