# Supplementary material for: A metagenomic viral discovery approach identifies potential zoonotic and novel mammalian viruses in Neoromicia bats within South Africa
Source: PLoS One. 2018 Mar 26;13(3):e0194527. doi: 10.1371/journal.pone.0194527 (PMC5868816; doi:10.1371/journal.pone.0194527)
Supplement: S6 Table — The table shows pairwise sequence similarities inferred from evolutionary divergence estimates of 249 positions of compared Bunyavirales. The number of base differences per site from between sequences were converted to percentage of similarities. Standard errors for distance estimates are shown above the diagonal. Codon positions included were 1–3 as well as noncoding. Ambiguous positions were removed for each sequence pair as per pairwise deletion. Estimates were analysed in MEGA7 [41]. (PDF) [file pone.0194527.s007.pdf]

S6 Table: Pairwise similarities inferred from distance estimations of an L gene region between selected *Bunyavirales*

	Accession	Members of the order as comparison	1	2	3	4	5	6	7	8	9	10	11	12	13	14	15	16	17	18	19	20	21	22	23	24	25	26	27	28	29	30	31
1	this study	NBt/PhenuiV/Neo11304/RSA		0.027	0.027	0.027	0.027	0.027	0.027	0.027	0.027	0.027	0.027	0.027	0.027	0.027	0.027	0.029	0.030	0.031	0.032	0.031	0.031	0.032	0.031	0.031	0.032	0.032	0.030	0.031	0.030	0.030	0.030
2	KX886767	Bat/VNM/16715_38	68.7		0.004	0.004	0.004	0.004	0.005	0.006	0.006	0.008	0.008	0.008	0.008	0.006	0.006	0.028	0.029	0.030	0.031	0.030	0.030	0.030	0.030	0.030	0.032	0.032	0.031	0.030	0.030	0.029	0.030
3	KX886785	Bat/VNM/16845_8	68.3	99.6		0.000	0.000	0.000	0.004	0.004	0.004	0.007	0.008	0.007	0.007	0.004	0.004	0.028	0.029	0.030	0.031	0.030	0.030	0.031	0.030	0.030	0.032	0.032	0.031	0.030	0.030	0.029	0.030
4	KX886779	Bat/VNM/16715_84	68.3	99.6	100.0		0.00	0.000	0.004	0.004	0.004	0.007	0.008	0.007	0.007	0.004	0.004	0.028	0.029	0.030	0.031	0.030	0.030	0.031	0.030	0.030	0.032	0.032	0.031	0.030	0.030	0.029	0.030
5	KX886775	Bat/VNM/16715_70	68.3	99.6	100.0	100.0		0.000	0.004	0.004	0.004	0.007	0.008	0.007	0.007	0.004	0.004	0.028	0.029	0.030	0.031	0.030	0.030	0.031	0.030	0.030	0.032	0.032	0.031	0.030	0.030	0.029	0.030
6	KX886769	Bat/VNM/16715_45	68.3	99.6	100.0	100.0	100.0		0.004	0.004	0.004	0.007	0.008	0.007	0.007	0.004	0.004	0.028	0.029	0.030	0.031	0.030	0.030	0.031	0.030	0.030	0.032	0.032	0.031	0.030	0.030	0.029	0.030
7	KX886761	Bat/VNM/16715_14	68.3	99.2	99.6	99.6	99.6	99.6		0.005	0.006	0.008	0.009	0.008	0.008	0.005	0.005	0.028	0.029	0.030	0.031	0.030	0.030	0.031	0.030	0.030	0.032	0.032	0.031	0.030	0.030	0.029	0.030
8	KX886759	Bat/VNM/16715_13	67.9	99.2	99.6	99.6	99.6	99.6	99.2		0.005	0.008	0.009	0.008	0.008	0.006	0.006	0.028	0.029	0.030	0.031	0.030	0.030	0.031	0.031	0.031	0.032	0.032	0.031	0.031	0.030	0.028	0.029
9	KX886783	Bat/VNM/16715_88	67.9	99.2	99.6	99.6	99.6	99.6	99.2	99.2		0.008	0.009	0.008	0.008	0.005	0.005	0.028	0.029	0.030	0.031	0.030	0.030	0.031	0.030	0.030	0.032	0.032	0.031	0.030	0.030	0.029	0.030
10	KX886781	Bat/VNM/16715_86	67.9	98.4	98.8	98.8	98.8	98.8	98.4	98.4	98.4		0.004	0.000	0.000	0.008	0.008	0.028	0.029	0.030	0.031	0.030	0.030	0.031	0.030	0.030	0.032	0.032	0.031	0.030	0.030	0.029	0.029
11	KX886777	Bat/VNM/16715_77	68.3	98.0	98.4	98.4	98.4	98.4	98.0	98.0	98.0	99.6		0.004	0.004	0.008	0.008	0.028	0.029	0.030	0.031	0.030	0.030	0.031	0.030	0.030	0.032	0.032	0.031	0.030	0.030	0.029	0.029
12	KX886773	Bat/VNM/16715_53	67.9	98.4	98.8	98.8	98.8	98.8	98.4	98.4	98.4	100.0	99.6		0.000	0.008	0.008	0.028	0.029	0.030	0.031	0.030	0.030	0.031	0.030	0.030	0.032	0.032	0.031	0.030	0.030	0.029	0.029
13	KX886765	Bat/VNM/16715_23	67.9	98.4	98.8	98.8	98.8	98.8	98.4	98.4	98.4	100.0	99.6	100.0		0.008	0.008	0.028	0.029	0.030	0.031	0.030	0.030	0.031	0.030	0.030	0.032	0.032	0.031	0.030	0.030	0.029	0.029
14	KX886763	Bat/VNM/16715_22	67.9	99.2	99.6	99.6	99.6	99.6	99.2	99.2	99.2	98.4	98.0	98.4	98.4		0.000	0.028	0.029	0.030	0.031	0.030	0.030	0.031	0.030	0.030	0.032	0.032	0.031	0.030	0.030	0.029	0.030
15	KX886771	Bat/VNM/16715_5	67.9	99.2	99.6	99.6	99.6	99.6	99.2	99.2	99.2	98.4	98.0	98.4	98.4	100.0		0.028	0.029	0.030	0.031	0.030	0.030	0.031	0.030	0.030	0.032	0.032	0.031	0.030	0.030	0.029	0.030
16	KC154063	Bat/Rh. pearsoni/Shaanxi/2011	63.5	70.3	69.9	69.9	69.9	69.9	69.9	70.3	69.5	69.5	69.1	69.5	69.5	70.3	70.3		0.030	0.030	0.030	0.031	0.031	0.031	0.030	0.030	0.030	0.031	0.030	0.030	0.030	0.029	0.030
17	KM817705	Zhee Mosquito virus XC1_8	61.0	61.4	61.8	61.8	61.8	61.8	61.4	62.2	61.4	61.4	61.8	61.4	61.4	61.4	61.4	60.6		0.030	0.030	0.030	0.030	0.031	0.029	0.029	0.030	0.031	0.030	0.029	0.030	0.029	0.030
18	NC_018463	Shamonda virus	38.2	43.4	43.4	43.4	43.4	43.4	43.4	43.8	43.4	43.8	43.8	43.8	43.8	43.4	43.4	38.2	37.3		0.027	0.028	0.027	0.029	0.029	0.029	0.030	0.030	0.030	0.029	0.029	0.030	0.030
19	KP691606	Oropouche virus BeH759021	39.8	43.4	43.0	43.0	43.0	43.0	43.0	43.4	43.4	41.8	41.4	41.8	41.8	43.4	43.4	43.0	37.8	73.5		0.027	0.027	0.028	0.029	0.029	0.031	0.031	0.030	0.029	0.030	0.030	0.030
20	KP063898	Bunyamwera virus SFAbCrEq238	41.8	41.8	42.2	42.2	42.2	42.2	42.2	42.6	42.2	41.0	41.0	41.0	41.0	42.6	42.6	43.0	39.4	70.3	72.3		0.026	0.027	0.029	0.029	0.031	0.031	0.030	0.029	0.030	0.030	0.029
21	NC_034499	Kaeng Khoi bat virus PSC-19	38.6	41.0	40.6	40.6	40.6	40.6	40.6	41.0	41.0	40.2	40.2	40.2	40.2	41.0	41.0	41.4	37.8	69.9	73.9	75.1		0.026	0.029	0.029	0.031	0.031	0.032	0.029	0.031	0.030	0.030
22	NC_034479	Bwamba virus M459	42.2	41.0	41.4	41.4	41.4	41.4	41.4	41.8	41.0	41.8	41.8	41.8	41.8	41.4	41.4	41.4	38.6	66.3	73.5	71.9	74.3		0.029	0.029	0.030	0.031	0.030	0.029	0.030	0.030	0.030
23	KR017842	SFTSV_HN-LR/China/05/2012	47.0	43.0	43.0	43.0	43.0	43.0	43.0	43.4	43.4	42.2	42.2	42.2	42.2	43.0	43.0	41.8	43.0	37.3	38.2	41.0	40.2	39.8		0.004	0.029	0.028	0.029	0.000	0.030	0.028	0.029
24	KU361343	SFTSV ZL13-32	46.6	43.4	43.4	43.4	43.4	43.4	43.4	43.8	43.8	42.6	42.6	42.6	42.6	43.4	43.4	41.8	42.6	37.8	38.2	41.4	40.2	39.8	99.6		0.028	0.028	0.029	0.004	0.030	0.028	0.029
25	KF186494	Malsoor virus NIV1050639	45.8	48.6	48.6	48.6	48.6	48.6	48.2	48.2	49.0	48.6	48.6	48.6	48.6	48.6	48.6	43.4	44.2	39.4	39.0	44.2	42.2	39.4	67.1	67.5		0.007	0.030	0.029	0.030	0.028	0.029
26	KF186497	Malsoor virus NIV1050650	46.2	48.2	48.2	48.2	48.2	48.2	47.8	47.8	48.6	48.2	48.2	48.2	48.2	48.2	48.2	43.0	44.6	39.8	39.0	44.6	42.6	39.8	67.9	68.3	98.8		0.030	0.028	0.030	0.028	0.029
27	KM817665	Changping Tick virus 1/CP1/2	49.0	47.4	47.4	47.4	47.4	47.4	47.8	47.8	47.0	47.8	47.8	47.8	47.8	47.0	47.0	44.2	46.2	37.8	37.3	39.8	41.8	41.4	53.4	53.4	49.0	49.4		0.029	0.030	0.029	0.029
28	JF906039	Huaiyangshan virus isolate XCQ-182L	47.0	43.0	43.0	43.0	43.0	43.0	43.0	43.4	43.4	42.2	42.2	42.2	42.2	43.0	43.0	41.8	43.0	37.3	38.2	41.0	40.2	39.8	100.0	99.6	67.1	67.9	53.4		0.030	0.028	0.029
29	NC_015374	Candiru virus	49.0	54.2	54.2	54.2	54.2	54.2	53.8	54.6	54.6	54.2	54.2	54.2	54.2	53.8	53.8	52.2	48.2	43.0	43.8	44.6	45.4	46.2	50.2	50.2	50.6	51.0	60.2	50.2		0.029	0.030
30	JQ287716	Mouyassue virus (hantavirus)	32.9	35.3	34.9	34.9	34.9	34.9	34.9	34.5	34.9	35.3	35.3	35.3	35.3	35.3	35.3	38.6	34.5	40.2	39.0	39.0	40.6	38.6	30.9	30.9	32.9	32.1	32.9	30.9	36.5		0.028
31	KF704717	Xuan son virus F44601 (hantavirus)	37.3	37.3	37.3	37.3	37.3	37.3	37.3	36.9	37.3	36.5	36.5	36.5	36.5	37.8	37.8	37.8	36.1	40.2	43.0	40.2	47.8	41.0	30.1	30.1	30.5	30.9	34.5	30.1	36.5	69.9	
