# Supplementary material for: A metagenomic viral discovery approach identifies potential zoonotic and novel mammalian viruses in Neoromicia bats within South Africa
Source: PLoS One. 2018 Mar 26;13(3):e0194527. doi: 10.1371/journal.pone.0194527 (PMC5868816; doi:10.1371/journal.pone.0194527)
Supplement: S7 Table — The table shows pairwise sequence similarities inferred from evolutionary divergence estimates of 605 positions of compared coronaviruses. The number of base differences per site from between sequences were converted to percentage of similarities. The sequences from this study are highlighted in grey and closest similarities to sequences from other studies are indicated in bold. Standard errors for distance estimates are shown above the diagonal in grey text. Codon positions included were 1–3 as well as noncoding and ambiguous positions were removed for each sequence pair as per pairwise deletion. Estimates were analysed in MEGA 7 [41]. (PDF) [file pone.0194527.s008.pdf]

S7 Table: Pairwise similarities inferred from distance estimations of a 605bp conserved segment of the coronavirus RNA dependent RNA polymerase gene

	Accession	Coronaviruses as comparison	1	2	3	4	5	6	7	8	9	10	11	12	13	14	15	16	17	18	19	20	21	22	23	24	25	26	27	28	29	30	31	32	33	34	35	36	37	38	39	40	Genera
1	NC_005831	HCoVNL63		0.018	0.017	0.018	0.018	0.016	0.015	0.016	0.017	0.017	0.018	0.017	0.016	0.017	0.017	0.016	0.017	0.017	0.017	0.017	0.018	0.019	0.019	0.019	0.019	0.019	0.019	0.019	0.020	0.019	0.020	0.020	0.020	0.020	0.020	0.019	0.019	0.019	0.019	0.019	
2	NC_002645	HCoV229E	73.72		0.013	0.010	0.010	0.017	0.017	0.018	0.017	0.018	0.017	0.018	0.017	0.017	0.017	0.018	0.018	0.018	0.018	0.018	0.017	0.019	0.019	0.020	0.020	0.020	0.020	0.020	0.020	0.020	0.020	0.020	0.020	0.020	0.020	0.019	0.019	0.020	0.020	0.019	
3	FJ710045	BtCoV*Hipposideros*/GhanaKwam/8/2008	77.52	87.44		0.012	0.012	0.017	0.017	0.017	0.017	0.018	0.017	0.018	0.017	0.017	0.018	0.017	0.018	0.018	0.018	0.018	0.017	0.019	0.019	0.020	0.020	0.020	0.019	0.019	0.020	0.020	0.020	0.020	0.020	0.020	0.020	0.019	0.019	0.019	0.020	0.019	
4	KY073747	BtCoV*Hipposideros* KY229E-1	73.72	92.40	89.42		0.009	0.017	0.017	0.017	0.017	0.019	0.017	0.018	0.016	0.016	0.017	0.018	0.019	0.018	0.018	0.018	0.017	0.019	0.019	0.020	0.020	0.020	0.020	0.020	0.019	0.019	0.020	0.020	0.020	0.020	0.020	0.019	0.019	0.020	0.020	0.019	
5	KY073748	BtCoV*Hipposideros* KY229E-8	73.88	92.56	90.25	94.21		0.017	0.017	0.018	0.018	0.018	0.017	0.018	0.017	0.017	0.017	0.017	0.018	0.018	0.018	0.018	0.018	0.019	0.019	0.019	0.019	0.019	0.020	0.020	0.019	0.019	0.019	0.019	0.019	0.019	0.019	0.019	0.019	0.020	0.020	0.020	
6	This study	BtCoV*Neoromicia* 1787/LP/RSA/2013	79.17	72.07	72.89	72.23	73.06		0.017	0.017	0.017	0.017	0.017	0.013	0.016	0.016	0.016	0.016	0.015	0.016	0.016	0.015	0.017	0.019	0.018	0.019	0.019	0.019	0.019	0.019	0.019	0.019	0.019	0.019	0.019	0.019	0.019	0.018	0.018	0.020	0.019	0.018	
7	NC_032107	BtCoV/*Triaenops afer*/KYNL63-9a	84.63	74.71	75.37	74.88	75.37	77.69		0.016	0.016	0.018	0.018	0.016	0.017	0.017	0.016	0.017	0.017	0.017	0.017	0.016	0.017	0.019	0.019	0.019	0.019	0.019	0.019	0.019	0.020	0.019	0.020	0.020	0.020	0.020	0.020	0.019	0.019	0.020	0.020	0.019	
8	KY073745	BtCoV/*Triaenops afer*/KYNL63-9b	76.20	71.57	74.05	72.56	72.40	73.88	79.17		0.004	0.018	0.018	0.017	0.017	0.017	0.017	0.017	0.018	0.017	0.017	0.018	0.017	0.019	0.019	0.019	0.019	0.019	0.019	0.020	0.019	0.019	0.019	0.019	0.019	0.019	0.019	0.020	0.020	0.020	0.020	0.019	
9	KY073746	BtCoV/*Triaenops afer*/KYNL63-15	76.36	71.90	74.21	72.23	72.56	73.55	79.01	99.01		0.018	0.017	0.017	0.017	0.017	0.017	0.017	0.018	0.017	0.017	0.017	0.017	0.019	0.019	0.019	0.019	0.019	0.019	0.020	0.020	0.019	0.019	0.019	0.019	0.019	0.019	0.020	0.020	0.020	0.020	0.019	
10	NC_003436	PEDV (Porcine)	73.22	70.58	70.74	70.74	71.07	74.05	71.24	73.72	74.55		0.019	0.018	0.018	0.018	0.017	0.018	0.018	0.016	0.016	0.018	0.018	0.019	0.019	0.019	0.019	0.019	0.019	0.019	0.020	0.020	0.020	0.020	0.020	0.020	0.020	0.019	0.019	0.020	0.019	0.019	
11	NC_002306	FIPV (Feline)	70.91	70.74	71.90	70.25	70.91	72.23	71.07	70.41	70.58	66.61		0.017	0.019	0.019	0.018	0.019	0.017	0.017	0.017	0.017	0.017	0.018	0.019	0.019	0.019	0.019	0.018	0.018	0.018	0.018	0.019	0.019	0.019	0.019	0.019	0.020	0.020	0.020	0.019	0.019	*Alphacoronavirus*
12	GU190239	BtCoV*Nyctalus*/BNM98-30/BGR/2008	75.54	71.74	72.23	72.23	73.22	85.79	75.87	74.88	74.38	73.39	70.74		0.016	0.016	0.016	0.017	0.015	0.016	0.016	0.015	0.018	0.018	0.018	0.020	0.020	0.020	0.019	0.019	0.019	0.019	0.019	0.019	0.019	0.019	0.019	0.019	0.019	0.020	0.019	0.019	
13	GU190247	BtCoV*Miniopterus*/BR98-52/BGR/2008	77.52	74.38	74.21	74.38	73.55	79.01	75.54	75.21	75.70	72.40	70.58	77.52		0.004	0.015	0.010	0.016	0.017	0.017	0.016	0.017	0.018	0.018	0.019	0.019	0.019	0.019	0.019	0.020	0.020	0.020	0.020	0.020	0.020	0.020	0.020	0.020	0.019	0.019	0.019	
14	GU190241	BtCoV*Miniopterus/*BR98-14/BGR/2008	76.86	73.88	74.05	73.88	73.39	79.01	75.54	74.55	75.04	72.40	70.58	77.36	99.01		0.015	0.010	0.015	0.017	0.017	0.015	0.017	0.018	0.018	0.019	0.019	0.019	0.019	0.019	0.019	0.020	0.019	0.019	0.019	0.019	0.019	0.020	0.020	0.019	0.019	0.019	
15	NC_010437	BtCoV*Miniopterus*/1A	75.37	72.56	73.22	74.05	74.38	79.01	76.69	74.38	74.05	74.21	71.57	77.02	80.00	79.83		0.016	0.016	0.016	0.016	0.016	0.016	0.019	0.018	0.019	0.020	0.019	0.019	0.019	0.019	0.019	0.020	0.020	0.020	0.020	0.020	0.019	0.019	0.020	0.020	0.019	
16	NC_010438	BtCoV*Miniopterus*/HKU8	77.85	74.21	73.55	74.38	73.22	77.19	75.21	76.03	75.87	73.39	71.24	75.87	93.72	93.39	80.17		0.016	0.017	0.017	0.016	0.017	0.019	0.019	0.020	0.020	0.020	0.019	0.019	0.019	0.019	0.019	0.019	0.019	0.019	0.019	0.019	0.019	0.019	0.019	0.019	
17	DQ249226	BtCoVHKU7-1	74.71	71.40	72.40	72.40	73.39	78.51	74.38	74.55	74.55	73.88	73.22	79.50	80.17	80.17	80.83	80.50		0.016	0.016	0.005	0.017	0.019	0.018	0.019	0.019	0.019	0.018	0.018	0.019	0.019	0.019	0.019	0.019	0.019	0.019	0.020	0.019	0.019	0.019	0.019	
18	KF294377	BtCoV*Murina*/Neixiang-14	74.38	71.24	71.74	70.74	72.23	78.18	76.36	72.73	72.89	76.69	70.91	75.54	75.04	75.21	76.53	75.37	75.87		0.000	0.016	0.016	0.019	0.018	0.018	0.018	0.018	0.019	0.019	0.019	0.019	0.019	0.019	0.019	0.019	0.019	0.019	0.019	0.020	0.019	0.019	
19	KF294376	BtCoV*Murina*/Neixiang-27	74.38	71.24	71.74	70.74	72.23	78.18	76.36	72.73	72.89	76.69	70.91	75.54	75.04	75.21	76.53	75.37	75.87	100.00		0.016	0.016	0.019	0.018	0.018	0.018	0.018	0.019	0.019	0.019	0.019	0.019	0.019	0.019	0.019	0.019	0.019	0.019	0.020	0.019	0.019	
20	KF294271	BtCoV*Miniopterus*/Anlong-36	74.38	71.07	72.23	71.74	73.06	79.34	74.71	74.55	74.55	73.06	74.05	80.17	80.33	80.66	81.16	80.66	98.18	75.70	75.70		0.017	0.019	0.018	0.019	0.019	0.019	0.018	0.018	0.019	0.019	0.019	0.019	0.019	0.019	0.019	0.020	0.019	0.019	0.019	0.019	
21	NC_009988	BtCoV*Rhinolophus*/HKU2	73.39	71.57	73.39	72.56	72.73	73.72	72.89	74.55	74.38	70.08	72.73	73.06	76.20	76.53	76.36	75.54	75.37	75.04	75.04	75.37		0.019	0.018	0.019	0.019	0.018	0.019	0.019	0.019	0.019	0.019	0.019	0.019	0.019	0.019	0.020	0.021	0.020	0.018	0.019	
22	NC_006577	HCoVHKU1	63.47	63.64	65.62	62.81	64.46	63.80	62.98	59.67	59.67	58.35	64.79	62.81	63.80	63.97	63.14	62.31	63.31	64.63	64.63	62.64	62.81		0.013	0.018	0.018	0.018	0.018	0.018	0.018	0.018	0.018	0.018	0.018	0.018	0.018	0.018	0.019	0.018	0.019	0.019	
23	NC_005147	HCoVOC43	62.98	63.31	63.64	62.31	63.80	60.66	63.47	60.83	60.50	57.36	61.32	60.33	63.14	62.98	60.50	62.48	62.98	62.64	62.64	62.15	62.15	85.12		0.018	0.018	0.018	0.018	0.018	0.018	0.018	0.018	0.018	0.018	0.018	0.018	0.019	0.019	0.019	0.019	0.018	
24	NC_004718	HCoV/SARS	61.32	61.16	62.81	61.49	62.31	62.15	62.31	61.16	61.16	61.98	60.17	61.82	62.31	61.98	63.80	61.65	63.14	64.46	64.46	62.31	63.97	66.61	66.28		0.005	0.004	0.019	0.018	0.018	0.018	0.019	0.019	0.019	0.019	0.019	0.020	0.020	0.019	0.019	0.020	
25	DQ071615	BtCoV*Rhinolophus* SARS-related Rp3	60.83	60.83	62.48	61.16	61.98	62.15	62.15	60.99	60.99	61.32	59.67	61.82	62.31	61.98	63.47	60.99	63.47	64.46	64.46	63.14	63.47	65.95	65.62	98.18		0.006	0.019	0.018	0.018	0.018	0.019	0.019	0.019	0.019	0.019	0.020	0.020	0.019	0.019	0.020	
26	KF367457	BtCoV*Rhinolophus* SARS-related WIV1	61.82	61.16	63.31	61.82	62.64	61.82	62.48	61.32	61.32	61.82	59.83	61.82	61.98	61.65	63.47	61.32	62.48	64.13	64.13	61.65	63.64	66.45	66.28	99.17	97.69		0.018	0.018	0.019	0.018	0.018	0.018	0.018	0.018	0.018	0.020	0.020	0.019	0.019	0.020	
27	NC_009019	BtCoV*Tylonycteris*/HKU4-1	61.49	61.82	63.14	61.82	62.81	61.65	60.99	59.67	59.17	60.83	66.94	62.98	62.81	62.64	65.45	63.47	63.97	63.64	63.64	63.80	62.64	69.59	68.26	67.77	67.60	67.93		0.014	0.016	0.016	0.016	0.016	0.016	0.016	0.016	0.018	0.018	0.019	0.019	0.019	
28	NC_009020	BtCoV*Pipistrellus*/HKU5-1	60.50	61.82	61.49	61.16	61.32	61.49	60.66	60.00	59.67	61.98	66.28	62.48	62.31	62.15	63.31	63.14	63.80	61.16	61.16	64.13	63.31	67.11	66.45	67.27	67.11	67.11	83.47		0.014	0.014	0.015	0.015	0.015	0.015	0.015	0.019	0.018	0.019	0.019	0.019	
29	this study	BtCoV*Neoromicia* 5038/KZN/RSA/2015	61.82	61.98	63.47	62.64	61.98	63.97	64.46	62.15	62.15	62.48	66.12	61.65	62.15	61.98	65.29	64.30	64.79	64.30	64.30	65.12	61.82	67.60	67.77	68.93	68.60	68.43	80.66	84.46		0.006	0.010	0.010	0.010	0.010	0.010	0.018	0.018	0.020	0.019	0.019	*Betacoronavirus*
30	KC869678	BtCoV*Neoromicia*/PML-PHE1/RSA/2011	61.96	61.13	62.79	61.79	61.13	63.46	63.29	61.30	61.30	61.46	66.11	60.80	60.63	60.80	64.12	63.62	63.62	63.62	63.62	64.12	62.13	67.61	66.94	68.60	68.44	68.11	79.57	83.72	97.67		0.010	0.010	0.010	0.010	0.010	0.019	0.019	0.020	0.019	0.019	
31	KF958702	HCoVMERS/Jeddah-1	60.99	61.32	62.98	62.31	61.65	63.31	61.98	60.99	60.99	62.31	64.96	61.49	61.49	61.32	63.80	62.64	64.46	63.14	63.14	64.30	61.32	65.95	65.79	67.11	67.60	67.27	79.34	83.80	93.55	93.36		0.000	0.000	0.000	0.000	0.018	0.018	0.020	0.019	0.019	
32	KF917527	CamelCoVMERS/Jeddah-1	60.99	61.32	62.98	62.31	61.65	63.31	61.98	60.99	60.99	62.31	64.96	61.49	61.49	61.32	63.80	62.64	64.46	63.14	63.14	64.30	61.32	65.95	65.79	67.11	67.60	67.27	79.34	83.80	93.55	93.36	100.00		0.000	0.000	0.000	0.018	0.018	0.020	0.019	0.019	
33	KJ713297	CamelCoVMERS/KSA-CAMEL-503	60.99	61.32	62.98	62.31	61.65	63.31	61.98	60.99	60.99	62.31	64.96	61.49	61.49	61.32	63.80	62.64	64.46	63.14	63.14	64.30	61.32	65.95	65.79	67.11	67.60	67.27	79.34	83.80	93.55	93.36	100.00	100.00		0.000	0.000	0.018	0.018	0.020	0.019	0.019	
34	KC164505	HCoVMERSEngland1	60.99	61.32	62.98	62.31	61.65	63.31	61.98	60.99	60.99	62.31	64.96	61.49	61.49	61.32	63.80	62.64	64.46	63.14	63.14	64.30	61.32	65.95	65.79	67.11	67.60	67.27	79.34	83.80	93.55	93.36	100.00	100.00	100.00		0.000	0.018	0.018	0.020	0.019	0.019	
35	KU308549	HCoVMERSKorea/Seoul/SNU1-035/2015	60.99	61.32	62.98	62.31	61.65	63.31	61.98	60.99	60.99	62.31	64.96	61.49	61.49	61.32	63.80	62.64	64.46	63.14	63.14	64.30	61.32	65.95	65.79	67.11	67.60	67.27	79.34	83.80	93.55	93.36	100.00	100.00	100.00	100.00		0.018	0.018	0.020	0.019	0.019	
36	NC_001451	AIBV	63.64	63.97	64.63	64.96	66.28	65.79	65.12	60.99	61.16	63.80	63.97	61.98	62.81	62.81	65.62	62.98	65.29	64.46	64.46	65.45	60.66	65.12	63.47	63.47	63.80	63.14	67.60	64.79	66.94	66.78	66.28	66.28	66.28	66.28	66.28		0.009	0.019	0.019	0.019	
37	NC_010800	TurkeyCoV	64.13	63.31	64.96	63.80	64.96	64.30	64.13	61.82	61.98	64.46	63.80	62.48	63.64	63.31	64.30	63.80	65.79	64.13	64.13	65.95	60.00	64.96	64.30	62.81	63.47	62.48	67.11	65.95	67.60	67.11	66.78	66.78	66.78	66.78	66.78	93.55		0.019	0.019	0.018	*Gammacoronavirus*
38	NC_010646	Beluga Whale CoV SW1	60.99	62.15	63.14	62.31	62.48	59.67	59.67	59.83	59.34	60.17	60.17	57.52	60.17	60.17	61.65	61.98	60.66	60.33	60.33	60.17	59.01	62.15	63.64	59.50	59.01	59.17	62.48	62.15	61.82	61.79	61.65	61.65	61.65	61.65	61.65	65.62	66.12		0.019	0.019	
39	FJ376620	Bulbul CoV HKU11-796	62.62	59.80	60.63	60.47	59.97	61.13	60.13	58.80	58.47	58.97	60.30	58.97	61.79	61.46	59.47	62.79	59.63	60.63	60.63	59.47	59.80	62.96	63.79	64.78	64.62	64.78	64.29	64.12	62.29	61.94	62.29	62.29	62.29	62.29	62.29	63.95	62.96	62.62		0.014	*Deltacoronavirus*
40	NC_011549	Thrush CoV HKU12-600	62.96	61.30	61.79	61.96	62.29	59.80	61.46	57.48	57.48	57.97	63.29	57.31	60.63	59.97	59.80	62.29	60.13	59.30	59.30	59.63	60.13	64.12	65.45	60.30	59.80	60.47	63.46	63.29	63.12	63.61	64.12	64.12	64.12	64.12	64.12	65.78	65.95	62.96	86.54		
