# Supplementary material for: A metagenomic viral discovery approach identifies potential zoonotic and novel mammalian viruses in Neoromicia bats within South Africa
Source: PLoS One. 2018 Mar 26;13(3):e0194527. doi: 10.1371/journal.pone.0194527 (PMC5868816; doi:10.1371/journal.pone.0194527)
Supplement: S8 Table — The percentage similarities were inferred from pairwise distance estimates of the base pair and amino acid differences per site for each gene and of the overall genome. The estimates were calculated in MEGA7 [41] using pairwise deletion to treat gaps. All percentage similarities are given in comparison to BtCoVNeoV5038. Accession number of compared betacoronaviruses are listed in order: KC869678.4, KX574227, EF065505.1, EF065509.1, JX869059.2, KF958702.1, KF917527.1, and KJ477102.1. (PDF) [file pone.0194527.s009.pdf]

S8 Table: Genome annotation of BtCoVNeo5038 with similarities to compared lineage C betacoronaviruses

Genome description of BtCoVNeo5038			Percentage identities to lineage C coronaviruses															
Open reading frames	Coding region position	No. of amino acids	*Neoromicia* NeoCoV PML/PHE1		*Pipistrellus* PREDICT-PDF2180		*Tylonycteris* BtCoV-HKU4		*Pipistrellus* BtCoV-HKU5		Human MERS EMC 2012		Human MERS Jeddah		Camel MERS Jeddah		Camel MERS NRCE HKU205	
			nt%	aa%	nt%	aa%	nt%	aa%	nt%	aa%	nt%	aa%	nt%	aa%	nt%	aa%	nt%	aa%
**Nonstructural genes**																		
**ORF1a**	268-13227	4319	96.9	98.1	85.4	89.4	66.9	64.5	66.9	66.0	86.2	89.9	89.8	89.8	86.1	89.8	86.2	89.8
**ORF1ab**	268..13440, 13440..21524	7085	97.2	98.6	87.3	86.2	72.0	73.4	72.0	75.5	88.5	92.8	88.4	92.8	88.4	92.8	88.4	92.7
**Coronavirus structural genes**																		
**Spike**	21466-25500	1344	96.0	98.7	85.5	93.5	62.5	60.2	61.8	60.9	67.9	64.0	68.0	63.9	68.0	63.9	67.8	63.7
**Envelope**	27573-27821	82	98.0	98.8	91.2	92.7	75.1	74.4	76.3	73.2	92.4	90.2	92.4	90.2	92.4	90.2	92.4	90.2
**Membrane**	27836-28495	219	97.7	99.1	85.8	94.5	74.9	82.2	71.9	82.2	87.9	94.5	87.7	94.1	87.7	94.1	87.9	94.5
**Nucleoprotein**	28553-29797	414	98.3	99.5	87.4	90.6	72.2	75.4	72.6	74.3	87.4	92.0	87.4	92.0	87.4	92.0	87.2	91.5
**Accessory proteins (hypothetical) of lineage C betacoronaviruses**																		
**ORF3**	25515-25826	103	97.8	97.3	76.5	74.3	49.6	30.1	57.5	26.3	80.4	85.0	80.8	85.0	80.8	85.0	81.1	84.5
**ORF4a**	25835-26164	109	98.2	99.1	78.2	75.2	54.7	38.9	54.0	43.0	86.4	87.2	87.0	87.2	87.0	87.2	87.3	87.2
**ORF4b**	26040-26816	258	97.2	97.3	72.7	70.9	47.2	30.1	45.7	26.3	83.9	85.0	83.9	85.0	83.9	85.0	83.8	84.5
**ORF5**	26823-27497	224	97.2	97.3	75.4	73.7	59.3	45.5	61.9	56.3	84.8	88.4	84.7	88.4	84.7	88.4	84.1	87.1
**ORF8b**	28599-29198	199	98.0	94.0	87.7	72.4	74.5	52.3	77.0	53.2	92.6	82.1	92.9	82.1	92.9	82.1	-	-
**Overall genome**	30009 bp	-	97.2	98.5	86.2	91.0	69.4	69.4	69.5	71	85.5	88.2	85.4	88.2	85.4	88.1	85.4	88.1
