# Supplementary material for: A metagenomic viral discovery approach identifies potential zoonotic and novel mammalian viruses in Neoromicia bats within South Africa
Source: PLoS One. 2018 Mar 26;13(3):e0194527. doi: 10.1371/journal.pone.0194527 (PMC5868816; doi:10.1371/journal.pone.0194527)
Supplement: S9 Table — The table shows pairwise similarities inferred from evolutionary divergence estimates of betacoronavirus full genomes. The number of base differences per site between sequences were converted to percentage similarities. Within lineage similarities are indicated in shaded blocks. Standard errors for distance estimates are shown above the diagonal in grey text. Codon positions included were 1–3 as well as noncoding and ambiguous positions were removed for each sequence pair as per pairwise deletion. Estimates were analysed in MEGA 7 [41]. (PDF) [file pone.0194527.s010.pdf]

**S9 Table: Pairwise similarities inferred from distance estimations between betacoronavirus full genomes**

| | Accession | Coronaviruses as comparison | Lineage | 1 | 2 | 3 | 4 | 5 | 6 | 7 | 8 | 9 | 10 | 11 | 12 | 13 | 14 | 15 | 16 | 17 | 18 | 19 | 20 | 21 | 22 | 23 | 24 | 25 | 26 | 27 | 28 |
|---|---|---|---|---|---|---|---|---|---|---|---|---|---|---|---|---|---|---|---|---|---|---|---|---|---|---|---|---|---|---|---|
| 1 | NC00657 | HCoV HKU1 | | | 0.002 | 0.002 | 0.002 | 0.003 | 0.003 | 0.003 | 0.003 | 0.003 | 0.003 | 0.003 | 0.003 | 0.003 | 0.002 | 0.002 | 0.002 | 0.002 | 0.002 | 0.002 | 0.002 | 0.002 | 0.002 | 0.002 | 0.003 | 0.002 | 0.003 | 0.003 | 0.003 |
| 2 | NC_003045 | Bovine CoV | A | 72.7 | | 0.003 | 0.001 | 0.002 | 0.002 | 0.002 | 0.003 | 0.002 | 0.002 | 0.002 | 0.003 | 0.003 | 0.002 | 0.002 | 0.002 | 0.002 | 0.002 | 0.002 | 0.002 | 0.002 | 0.002 | 0.002 | 0.003 | 0.003 | 0.002 | 0.003 | 0.003 |
| 3 | NC_006852 | MHV-JHM | | 72.0 | 72.3 | | 0.003 | 0.002 | 0.002 | 0.002 | 0.002 | 0.002 | 0.003 | 0.002 | 0.003 | 0.003 | 0.002 | 0.003 | 0.003 | 0.003 | 0.003 | 0.003 | 0.003 | 0.003 | 0.003 | 0.003 | 0.003 | 0.002 | 0.003 | 0.003 | 0.003 |
| 4 | NC005147 | HCoV OC43 | | 72.6 | 96.6 | 72.5 | | 0.002 | 0.002 | 0.002 | 0.002 | 0.003 | 0.002 | 0.002 | 0.003 | 0.003 | 0.002 | 0.003 | 0.003 | 0.003 | 0.003 | 0.003 | 0.003 | 0.003 | 0.003 | 0.003 | 0.003 | 0.003 | 0.002 | 0.003 | 0.003 |
| 5 | AY714217 | HCoV SARS-CoV CDC | | 45.7 | 45.2 | 44.1 | 45.2 | | 0.000 | 0.000 | 0.002 | 0.001 | 0.002 | 0.001 | 0.002 | 0.003 | 0.002 | 0.002 | 0.002 | 0.002 | 0.002 | 0.002 | 0.002 | 0.002 | 0.002 | 0.002 | 0.002 | 0.002 | 0.003 | 0.002 | 0.002 |
| 6 | GU553365 | HCoV SARS-CoV HKU39849 | | 45.6 | 45.1 | 43.9 | 45.1 | 100.0 | | 0.000 | 0.002 | 0.001 | 0.002 | 0.001 | 0.002 | 0.003 | 0.002 | 0.002 | 0.002 | 0.002 | 0.002 | 0.002 | 0.002 | 0.002 | 0.002 | 0.002 | 0.002 | 0.002 | 0.003 | 0.002 | 0.003 |
| 7 | AY572034 | Civet SARS-CoV 007 | | 45.6 | 45.1 | 43.9 | 45.1 | 99.7 | 99.7 | | 0.002 | 0.001 | 0.002 | 0.001 | 0.002 | 0.003 | 0.002 | 0.002 | 0.002 | 0.002 | 0.002 | 0.002 | 0.002 | 0.002 | 0.002 | 0.002 | 0.002 | 0.002 | 0.003 | 0.002 | 0.002 |
| 8 | NC_009694 | BtCoV*Rhinolophus*-SARS HKU3 | B | 45.5 | 44.8 | 44.0 | 44.9 | 87.8 | 87.9 | 87.7 | | 0.002 | 0.002 | 0.001 | 0.002 | 0.002 | 0.002 | 0.002 | 0.002 | 0.002 | 0.002 | 0.002 | 0.002 | 0.002 | 0.002 | 0.002 | 0.003 | 0.002 | 0.003 | 0.003 | 0.003 |
| 9 | KF367457 | BtCoV*Rhinolophus*-SARS WIV1 | | 45.4 | 44.6 | 43.5 | 44.9 | 95.6 | 95.6 | 95.4 | 88.2 | | 0.002 | 0.001 | 0.002 | 0.003 | 0.003 | 0.002 | 0.002 | 0.002 | 0.002 | 0.002 | 0.002 | 0.002 | 0.002 | 0.002 | 0.002 | 0.002 | 0.003 | 0.002 | 0.002 |
| 10 | DQ412043 | BtCoV*Rhinolophus*-SARS Rm1 | | 45.2 | 44.5 | 43.7 | 44.7 | 88.1 | 88.1 | 88.0 | 90.3 | 88.5 | | 0.002 | 0.002 | 0.002 | 0.002 | 0.002 | 0.002 | 0.002 | 0.002 | 0.002 | 0.002 | 0.002 | 0.002 | 0.002 | 0.002 | 0.002 | 0.003 | 0.002 | 0.003 |
| 11 | DQ071615 | BtCoV*Rhinolophus*-SARS Rp3 | | 45.6 | 45.0 | 44.0 | 45.0 | 92.5 | 92.6 | 92.4 | 89.7 | 93.5 | 90.5 | | 0.003 | 0.002 | 0.002 | 0.002 | 0.002 | 0.002 | 0.002 | 0.002 | 0.002 | 0.002 | 0.002 | 0.002 | 0.002 | 0.002 | 0.002 | 0.002 | 0.002 |
| 12 | NC_009021 | BtCoV*Rousettus*/HKU9-1 | D | 46.2 | 45.4 | 44.0 | 45.4 | 51.8 | 51.8 | 51.8 | 51.5 | 51.6 | 51.5 | 51.7 | | 0.002 | 0.002 | 0.003 | 0.003 | 0.003 | 0.003 | 0.003 | 0.003 | 0.003 | 0.003 | 0.003 | 0.002 | 0.003 | 0.002 | 0.002 | 0.002 |
| 13 | EF065516 | BtCoV*Rousettus*/HKU9-4 | | 45.8 | 44.9 | 43.8 | 44.9 | 51.6 | 51.5 | 51.5 | 51.1 | 51.3 | 50.9 | 51.3 | 79.6 | | 0.003 | 0.003 | 0.003 | 0.003 | 0.003 | 0.003 | 0.003 | 0.003 | 0.003 | 0.003 | 0.003 | 0.003 | 0.003 | 0.003 | 0.002 |
| 14 | NC_022643 | *Erinaceus* CoV VMC/DEU/2012 | | 47.4 | 46.4 | 44.9 | 46.4 | 49.2 | 49.1 | 49.2 | 49.1 | 49.1 | 49.1 | 49.2 | 47.7 | 47.7 | | 0.003 | 0.002 | 0.002 | 0.002 | 0.002 | 0.002 | 0.002 | 0.003 | 0.002 | 0.003 | 0.002 | 0.002 | 0.002 | 0.002 |
| 15 | KC164505 | HCoVMERS/England1 | | 46.1 | 45.5 | 44.4 | 45.6 | 49.5 | 49.3 | 49.4 | 49.3 | 49.3 | 49.1 | 49.4 | 47.6 | 47.3 | 69.3 | | 0.000 | 0.000 | 0.000 | 0.000 | 0.000 | 0.000 | 0.000 | 0.000 | 0.003 | 0.002 | 0.002 | 0.001 | 0.002 |
| 16 | KM210277 | HCoVMERS/England/4/2013 | | 46.1 | 45.6 | 44.4 | 45.6 | 49.5 | 49.3 | 49.4 | 49.2 | 49.2 | 49.1 | 49.4 | 47.6 | 47.3 | 69.3 | 99.8 | | 0.000 | 0.000 | 0.000 | 0.000 | 0.000 | 0.000 | 0.000 | 0.003 | 0.002 | 0.002 | 0.001 | 0.002 |
| 17 | KU308549 | HCoVMERS/Korea/Seoul/SNU/103 | | 46.1 | 45.5 | 44.4 | 45.6 | 49.4 | 49.3 | 49.4 | 49.2 | 49.3 | 49.1 | 49.4 | 47.6 | 47.3 | 69.3 | 99.8 | 99.7 | | 0.000 | 0.000 | 0.000 | 0.000 | 0.000 | 0.000 | 0.003 | 0.002 | 0.002 | 0.001 | 0.002 |
| 18 | KF958702 | HCoVMERS/Jeddah/human/1 | | 46.2 | 45.6 | 44.4 | 45.7 | 49.5 | 49.4 | 49.4 | 49.3 | 49.4 | 49.2 | 49.5 | 47.7 | 47.4 | 69.2 | 99.9 | 99.8 | 99.7 | | 0.000 | 0.000 | 0.000 | 0.000 | 0.000 | 0.003 | 0.002 | 0.002 | 0.001 | 0.002 |
| 19 | KF917527 | HCoVMERS/Jeddah/Camel/1 | | 46.2 | 45.6 | 44.4 | 45.7 | 49.5 | 49.4 | 49.4 | 49.3 | 49.4 | 49.2 | 49.5 | 47.7 | 47.4 | 69.2 | 99.9 | 99.8 | 99.7 | 100.0 | | 0.000 | 0.000 | 0.000 | 0.000 | 0.003 | 0.002 | 0.002 | 0.001 | 0.002 |
| 20 | KJ713299 | HCoVMERS/KSA/CAMEL/376 | | 46.1 | 45.6 | 44.4 | 45.6 | 49.5 | 49.3 | 49.4 | 49.3 | 49.3 | 49.1 | 49.4 | 47.6 | 47.3 | 69.3 | 99.8 | 99.9 | 99.7 | 99.8 | 99.8 | | 0.000 | 0.000 | 0.000 | 0.003 | 0.002 | 0.002 | 0.001 | 0.002 |
| 21 | KJ713298 | HCoVMERS/KSA/CAMEL/363 | C | 46.1 | 45.6 | 44.4 | 45.6 | 49.5 | 49.3 | 49.4 | 49.2 | 49.2 | 49.1 | 49.4 | 47.6 | 47.3 | 69.3 | 99.9 | 99.8 | 99.7 | 99.8 | 99.8 | 99.8 | | 0.000 | 0.000 | 0.003 | 0.002 | 0.002 | 0.001 | 0.002 |
| 22 | KJ713297 | HCoVMERS/KSA/CAMEL/503 | | 46.1 | 45.6 | 44.4 | 45.6 | 49.5 | 49.3 | 49.4 | 49.2 | 49.2 | 49.1 | 49.4 | 47.6 | 47.3 | 69.3 | 99.8 | 99.8 | 99.7 | 99.8 | 99.8 | 99.8 | 99.9 | | 0.000 | 0.003 | 0.002 | 0.002 | 0.001 | 0.002 |
| 23 | KJ477102 | HCoVMERS/CAMEL/NRCE-HKU20 | | 46.1 | 45.5 | 44.4 | 45.6 | 49.5 | 49.4 | 49.4 | 49.2 | 49.3 | 49.0 | 49.4 | 47.6 | 47.3 | 69.2 | 99.5 | 99.5 | 99.3 | 99.4 | 99.4 | 99.4 | 99.5 | 99.4 | | 0.003 | 0.002 | 0.002 | 0.001 | 0.001 |
| 24 | NC009020 | BatCoV*Pipistrellus*/HKU5 | | 45.1 | 44.3 | 43.8 | 44.4 | 48.4 | 48.3 | 48.3 | 48.2 | 48.1 | 48.0 | 48.3 | 46.9 | 46.6 | 66.1 | 70.1 | 70.0 | 70.1 | 69.8 | 69.8 | 70.0 | 70.0 | 70.0 | 69.9 | | 0.003 | 0.002 | 0.002 | 0.003 |
| 25 | NC009019 | BatCoV*Tylonycteris*/HKU4 | | 47.4 | 46.3 | 44.7 | 46.3 | 49.6 | 49.4 | 49.5 | 49.2 | 49.3 | 49.1 | 49.6 | 48.2 | 47.5 | 67.1 | 69.7 | 69.6 | 69.7 | 69.5 | 69.5 | 69.7 | 69.7 | 69.7 | 69.5 | 72.9 | | 0.002 | 0.003 | 0.003 |
| 26 | KX574227 | BtCoV*Pipistrellus*/PREDICT-PDF2180 | | 46.9 | 45.8 | 45.0 | 45.8 | 49.4 | 49.4 | 49.4 | 49.2 | 49.2 | 49.2 | 49.4 | 48.0 | 47.5 | 70.0 | 83.4 | 83.4 | 83.4 | 83.4 | 83.4 | 83.4 | 83.4 | 83.4 | 83.3 | 69.7 | 69.3 | | 0.001 | 0.001 |
| 27 | KC869678 | BtCoV*Neoromicia*/PML/RSA/2011 | | 46.9 | 45.8 | 44.8 | 45.9 | 49.5 | 49.4 | 49.4 | 49.3 | 49.2 | 49.1 | 49.4 | 48.2 | 47.8 | 70.0 | 85.6 | 85.5 | 85.5 | 85.4 | 85.4 | 85.5 | 85.5 | 85.6 | 85.4 | 69.7 | 69.4 | 86.2 | | 0.001 |
| 28 | this study | BtCoV*Neoromicia* 5038/KZN/RSA/2015 | | 47.0 | 45.8 | 44.8 | 45.9 | 49.5 | 49.4 | 49.4 | 49.3 | 49.2 | 49.1 | 49.4 | 48.3 | 47.9 | 70.1 | 85.5 | 85.5 | 85.5 | 85.4 | 85.4 | 85.5 | 85.5 | 85.5 | 85.4 | 69.5 | 69.4 | 86.3 | 97.2 | |
