# Supplementary material for: A metagenomic viral discovery approach identifies potential zoonotic and novel mammalian viruses in Neoromicia bats within South Africa
Source: PLoS One. 2018 Mar 26;13(3):e0194527. doi: 10.1371/journal.pone.0194527 (PMC5868816; doi:10.1371/journal.pone.0194527)
Supplement: S10 Table — (PDF) [file pone.0194527.s011.pdf]

S10 Table: Coronavirus strains and Genbank accession numbers of sequences used in the full genome phylogeny.

Coronavirus	Abbreviation used	Genbank accession number
Human coronavirus OC43	HCoVOC43	NC005147
Bovine coronavirus	BovineCoV	NC_003045
Human coronavirus HKU1	HCoVHKU1	NC006577
Murine hepatitis virus strain JHM	MHV	NC_006852
Human SARS coronavirus CDC200301157	HCoVSARS	AY714217
Civet SARS coronavirus 007	HCoVCivet	AY572034
Human SARS coronavirus	HKU-39849	GU553365
Rhinolophus SARS-related coronavirus Rp3	Rp3	DQ071615
Rhinolophus SARS-related coronavirus Rm1	Rm1	DQ412043
Rhinolophus SARS-related coronavirus HKU3	HKU3	NC_009694
Rhinolophus SARS-related coronavirus WIV1	WIV1	KF367457
Rousettus coronavirus HKU9-1	HKU9	NC_009021
Rousettus coronavirus HKU9-4	HKU9	EF065516
Erinaceus CoV/2012-216/GER/2012	Erinaceus2012	NC_022643
Human MERS strain England 1	HCoVMERS	KC164505
Human MERS strain England/4/2013	HCoVMERS	KM210277
Human MERS strain Korea/Seoul/SNU1-035/2015	HCoVMERS	KU308549
Human MERS strain Jeddah-human-1	HCoVMERS	KF958702
MERS camel strain Jeddah-camel-1	Camel MERS	KF917527
MERS camel strain 376	Camel MERS	KJ713299
MERS camel strain 363	Camel MERS	KJ713298
MERS camel strain 503	Camel MERS	KJ713297
MERS camel strain NRCE-HKU205	Camel MERS	KJ477102
Neoromicia/PML-PHE1/RSA/2011	NeoCoV PML-PHE1	KC869678
Neoromicia bat coronavirus 5038/KZN/RSA/2015	BtCoVNeo5038	MF593268
Pipistrellus bat coronavirus HKU5	HKU5	NC009020
Tylonycteris bat coronavirus HKU4	HKU4	NC009019
